# Small Regulatory RNA-Induced Growth Rate Heterogeneity of *Bacillus subtilis*

**DOI:** 10.1371/journal.pgen.1005046

**Published:** 2015-03-19

**Authors:** Ruben A. T. Mars, Pierre Nicolas, Mariano Ciccolini, Ewoud Reilman, Alexander Reder, Marc Schaffer, Ulrike Mäder, Uwe Völker, Jan Maarten van Dijl, Emma L. Denham

**Affiliations:** 1 Department of Medical Microbiology, University of Groningen, University Medical Center Groningen, Groningen, the Netherlands; 2 INRA, UR1077, Mathématique Informatique et Génome, Jouy-en-Josas, France; 3 Interfaculty Institute for Genetics and Functional Genomics, Ernst-Moritz-Arndt-University Greifswald, Greifswald, Germany; Indiana University, UNITED STATES

## Abstract

Isogenic bacterial populations can consist of cells displaying heterogeneous physiological traits. Small regulatory RNAs (sRNAs) could affect this heterogeneity since they act by fine-tuning mRNA or protein levels to coordinate the appropriate cellular behavior. Here we show that the sRNA RnaC/S1022 from the Gram-positive bacterium *Bacillus subtilis* can suppress exponential growth by modulation of the transcriptional regulator AbrB. Specifically, the post-transcriptional *abrB*-RnaC/S1022 interaction allows *B*. *subtilis* to increase the cell-to-cell variation in AbrB protein levels, despite strong negative autoregulation of the *abrB* promoter. This behavior is consistent with existing mathematical models of sRNA action, thus suggesting that induction of protein expression noise could be a new general aspect of sRNA regulation. Importantly, we show that the sRNA-induced diversity in AbrB levels generates heterogeneity in growth rates during the exponential growth phase. Based on these findings, we hypothesize that the resulting subpopulations of fast- and slow-growing *B*. *subtilis* cells reflect a bet-hedging strategy for enhanced survival of unfavorable conditions.

## Author Summary

Bacterial cells that share the same genetic information can display very different phenotypes, even if they grow under identical conditions. Despite the relevance of this population heterogeneity for processes like drug resistance and development, the molecular players that induce heterogenic phenotypes are often not known. Here we report that in the Gram-positive model bacterium *Bacillus subtilis* a small regulatory RNA (sRNA) can induce heterogeneity in growth rates by increasing cell-to-cell variation in the levels of the transcriptional regulator AbrB, which is important for rapid growth. Remarkably, the observed variation in AbrB levels is induced post-transcriptionally because of AbrB’s negative autoregulation, and is not observed at the *abrB* promoter level. We show that our observations are consistent with mathematical models of sRNA action, thus suggesting that induction of protein expression noise could be a new general aspect of sRNA regulation. Since a low growth rate can be beneficial for cellular survival, we propose that the observed subpopulations of fast- and slow-growing *B*. *subtilis* cells reflect a bet-hedging strategy for enhanced survival of unfavorable conditions.

## Introduction

In their natural habitats, bacteria constantly adapt to changing environmental conditions while simultaneously anticipating further disturbances. To efficiently cope with these changes, intricate interlinked metabolic and genetic regulation has evolved [1]. This complex regulatory network includes the action of small regulatory RNAs (sRNAs) [2]. sRNAs are a widespread means for bacterial cells to coordinate (stress) responses by fine-tuning levels of mRNAs or proteins, and they have been studied in great detail in Gram-negative bacteria [3]. Regulation by some sRNAs takes place by short complementary base pairing to their target mRNA molecules, for instance in the region of the ribosome-binding site (RBS) to inhibit translation or trigger mRNA degradation. In Gram-negative bacteria many of these sRNA-mRNA interactions are mediated by the RNA chaperone Hfq [4]. However, the Hfq homologue in the Gram-positive model bacterium *Bacillus subtilis* has no effect on the regulation of the eight sRNA targets reported in this species so far [5–7]. Owing to the complexity of sRNA regulation, only a relatively small number of studies have focused specifically on the physiological necessity of sRNA-target interactions. This is again particularly true for Gram-positive bacteria, such as *B*. *subtilis*, despite the fact that many potential sRNAs have been identified [8, 9].

Within a bacterial population, genes and proteins can be expressed with a large variability, with high expression levels in some cells and low expression levels in others [10]. Examples of expression heterogeneity in *B*. *subtilis* are the extensively studied development of natural competence for DNA binding and uptake and the differentiation into spores [11–13]. In both cases, expression heterogeneity is generated by positive feedback loops, and results in bistable or ON-OFF expression of crucial regulators [14]. Distinctly from bistability, proteins can also be expressed with large cell-to-cell variability. This variation in expression levels, or noise, can originate from intrinsic or extrinsic sources [15, 16]. Extrinsic noise is related to cell-to-cell fluctuations in numbers of RNA polymerase, numbers of genome copies, or numbers of free ribosomes. Conversely, intrinsic noise is caused by factors directly involved in the transcription or translation of the respective gene or protein. Interestingly, particularly noisy genes are often found to be regulators of development and bacterial persistence [12, 17, 18]. Because of the importance of noise in protein expression, cells have evolved mechanisms to regulate the noise levels of at least some proteins [10]. Reducing noise levels has been suggested as an important explanation why many transcriptional regulators in bacteria (40% in *E*. *coli* [19]) autorepress the transcription of their own promoter (i.e. negative autoregulation (NAR)).

AbrB is a global transcriptional regulator in Gram-positive bacteria, including the important human pathogens *Bacillus anthracis* and *Listeria monocytogenes* [20, 21]. *B*. *subtilis* AbrB positively regulates some genes when carbon catabolite repression (CCR) is relieved [22], and negatively regulates the expression of over two hundred genes in the exponential growth phase [23]. Transcription of *abrB* is negatively autoregulated by binding of AbrB tetramers to the *abrB* promoter [24, 25]. Upon entry into stationary phase, *abrB* transcription is repressed via increasing levels of Spo0A-P and AbrB is inactivated by AbbA [26, 27]. The resulting AbrB depletion is consequently followed by activation of AbrB repressed genes, which are often important for stationary phase processes. Notably, because of its role in the elaborate sporulation and competence decision making network [26, 27], AbrB has mainly been studied in the context of entry into stationary phase while much less is known about its exact role in the exponential growth phase.

We selected putative sRNAs from a rich tiling array dataset of 1583 potentially regulatory RNAs [9]. This selection was made for evolutionary conserved putative sRNAs with a high expression level on defined minimal medium. Deletion strains of these putative *B*. *subtilis* sRNAs were subsequently tested for growth phenotypes. One sRNA—RnaC/S1022—stood out since the mutant strain displayed a strongly increased final optical density on minimal medium with sucrose as the sole carbon source. The present study was therefore aimed at determining how RnaC/S1022 influences the growth of *B*. *subtilis*. Inspection of consistently observed predicted RnaC/S1022 targets indicated that the aberrant growth phenotype could relate to elevated AbrB levels. Here we show that, under certain conditions, *B*. *subtilis* employs RnaC/S1022 to post-transcriptionally modulate AbrB protein expression noise. The observed noise in AbrB protein levels is remarkable, because the *abrB* gene displays low transcriptional noise consistent with its NAR. Importantly, the sRNA-induced noise in the AbrB protein levels generates growth rate heterogeneity in the exponential phase.

## Results

### RnaC/S1022 deletion enhances growth on minimal medium

**Fig 1:**
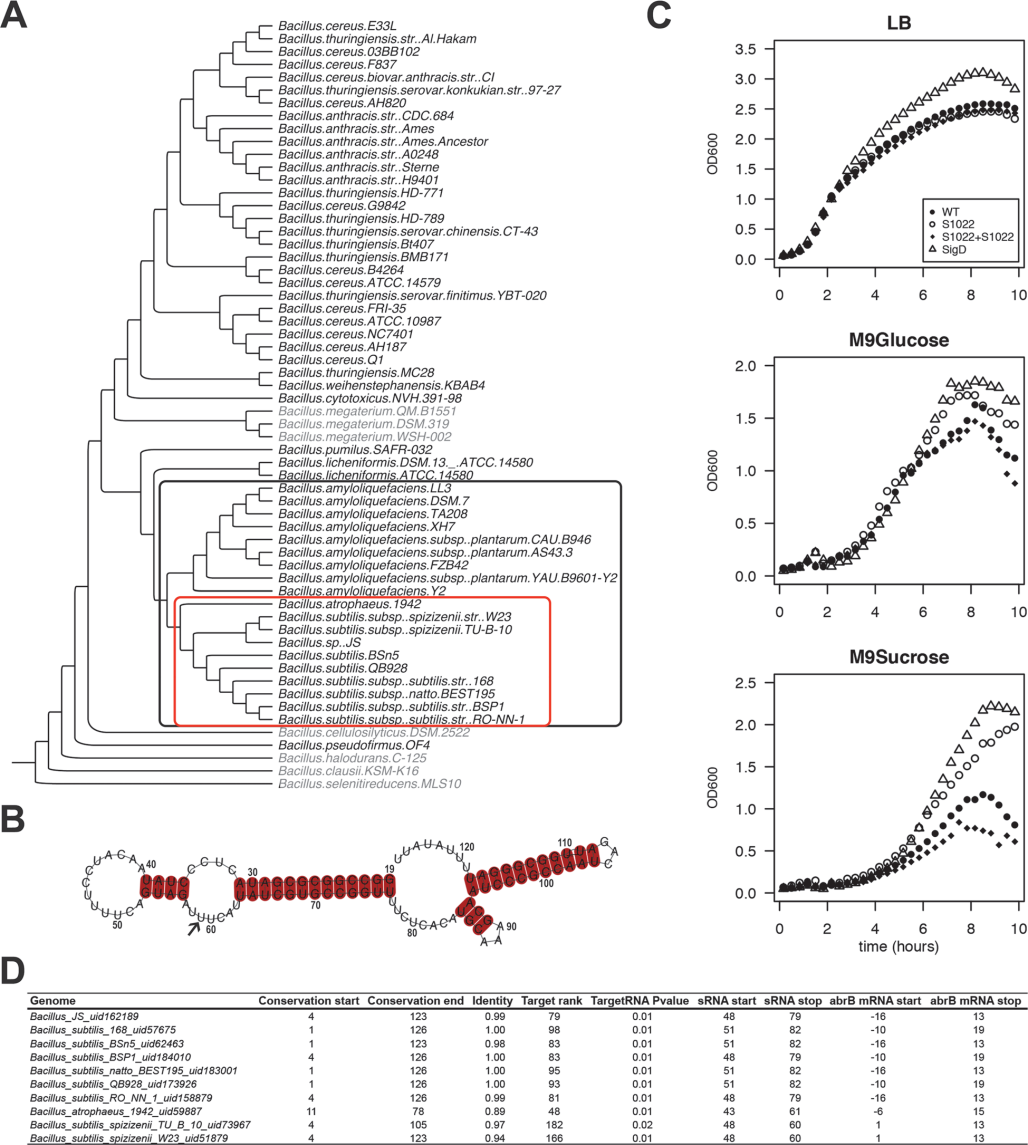
The RnaC/S1022 growth phenotype is linked to the evolutionary target prediction of abrB. A) Phylogenetic tree of *Bacillus* genomes. The tree was constructed based on an alignment of *rpoB* (present in 60 of the 62 genomes; except for two *Bacillus coagulans* genomes for which no significant *rpoB* nBLAST hits were found). The outer (black) box indicates 19 genomes in which RnaC/S1022 is present. The inner (red) box indicates 10 genomes in which the predicted RnaC/S1022-*abrB* interaction is consistently observed. A significant nBLAST hit for AbrB was not obtained for the species shaded in grey. B) LocARNA structural conservation alignment of RnaC/S1022 based on the sequence published by Schmalisch et al. [28]. The alignment includes RnaC/S1022 sequences from genomes in which the RnaC/S1022-*abrB* interaction is consistently observed (marked in the red box in panel A), except the RnaC/S1022 from *B*. *atrophaeus* (see also S1 Fig.). Numbers indicate the coordinates of S1022 defined by Nicolas et al. [9]. The arrow highlights the uracil base that is required for the interaction with *abrB*. C) Growth curves of parental strain 168^trp+^, ΔRnaC/S1022, Δ*sigD*, and the ΔRnaC/S1022 *amyE*::RnaC/S1022 complemented strain grown on LB, M9G or M9S. Each experiment was repeated at least three times in 96-well plates and shake flasks. Averages from triplicates from a representative 96-well plate experiment are shown. The OD_600_ was monitored every 10 min. One in two time-points were plotted. D) Overview of the predicted RnaC/S1022-*abrB* interaction. “Genome”, name of the bacterium in which the interaction was predicted. “Conservation start”, the base of the RnaC/S1022 sequence as defined by Nicolas et al. [9] where the significant nBLAST hit starts. “Conservation end”, end coordinate of the significant nBLAST hit. “Identity”, fraction of identity of the conserved RnaC/S1022 sequence compared to RnaC/S1022 of *B*. *subtilis* 168. “Target Rank” the ranking of the *abrB* target in the predicted RnaC/S1022 targets for the respective genome. “TargetRNA P value” TargetRNA_v1 prediction P-value. “sRNA_start”, start coordinate of RnaC/S1022 homologue in the predicted target interaction. “sRNA_stop”, end coordinate of RnaC/S1022 homologue in the predicted target interaction. “*abrB* mRNA_start”, start coordinate of *abrB* in the predicted target interaction relative to its start codon. “*abrB* mRNA_stop”, end coordinate of *abrB* in the predicted target interaction relative to its start codon.

RnaC/S1022 was first identified in a systematic screening of *B*. *subtilis* intergenic regions with an oligonucleotide microarray [28]. RnaC/S1022 is located in between *yrhK*, a gene of unknown function, and *cypB*, encoding cytochrome P450 NADPH-cytochrome P450 reductase (also known as *yrhJ*). We tested the conservation of the *B*. *subtilis* RnaC/S1022 sequence with BLAST analysis against a set of 62 *Bacillus* genomes, and found evolutionary conservation in a clade of the phylogenetic tree including 19 *B*. *subtilis*, *Bacillus atrophaeus*, and *Bacillus amyloliquefaciens* genomes (Fig. 1A and S1 Fig. for extensive alignments). Within these 19 genomes, the 5’ and 3’ ends of the RnaC/S1022 sequence are conserved, but the core sequence is disrupted in all 9 *B*. *amyloliquefaciens* genomes (S1 Fig.). Notably, the RnaC/S1022 from *B*. *atropheus* 1942 seems to represent an in-between form of RnaC/S1022 that mostly resembles the RnaC/S1022 sequences from the *B*. *amyloliquefaciens* sp. genomes. Therefore, an alignment of only the RnaC/S1022 sequences from the 9 remaining *B*. *subtilis* genomes was used to predict the RnaC/S1022 secondary structure using the LocARNA tool [29] (Fig. 1B, S1 Fig.). These analyses predict RnaC/S1022 to fold into a stable structure with a Gibbs free energy for the sequence shown in Fig. 1B of −38.5 kcal/mol, as calculated with RNAfold [30].

RnaC/S1022 was recently included in a screen for possible functions of conserved putative sRNAs identified by Nicolas et al. [9] that are highly expressed on M9 minimal medium supplemented with different carbon sources. Here, the RnaC/S1022 mutant stood out, because it consistently grew to a higher optical density (OD) in M9 minimal medium supplemented with sucrose (M9S) than the parental strain (Fig. 1C). To distinguish effects on the growth rate and growth yield, lin-log plots of these growth profiles are presented in S2 Fig., which show that the growth rate was only slightly influenced by the RnaC/S1022 deletion while the growth yield was strongly increased. Compared to M9S, the growth phenotype was less pronounced in M9 with glucose (M9G). Since transcription of RnaC/S1022 is exclusively regulated by SigD [28], we also tested a *sigD* mutant for growth under these conditions. Interestingly, the Δ*sigD* mutant displayed similar growth characteristics as the ΔRnaC/S1022 mutant (Fig. 1C). Differential growth and increased competitiveness were previously reported for a *sigD* mutant [31], and our observations suggest that in some conditions the increased final OD of the Δ*sigD* strain is partly due to deregulation of RnaC/S1022.

### AbrB is a consistently predicted target of RnaC/S1022

We wondered whether deregulation of an sRNA target was responsible for the remarkable growth phenotype observed for the ΔRnaC/S1022 mutant and decided to perform exploratory target predictions using TargetRNA [32]. Predicting sRNA targets can be successful, but target verification is complicated by the large number of false-positively predicted targets. We argued that additional information about the likelihood of a true target could be obtained by determining whether the predicted interaction is conserved over evolutionary time. To identify predicted RnaC/S1022-target interactions that are conserved, a bioinformatics pipeline was established that predicts sRNA targets in genomes in which the RnaC/S1022 sequence is conserved. Since we were interested in finding true *B*. *subtilis* sRNA targets, we only considered targets also predicted in *B*. *subtilis*, and these are listed in S1 Table. This analysis reduced the number of considered RnaC/S1022 targets to 47 (from 147 predicted targets for TargetRNA_v1 predictions with P value ≤ 0.01 on the *B*. *subtilis* 168 genome). These 47 predicted targets included seven sporulation-related genes (*phrA*, *spoVAD*, *spoIIM*, *spoIIIAG*, *cotO*, *sspG*, *spsI*). The sigma factor *sigM* was also consistently predicted but, since a *sigM* mutant strain only displays a growth phenotype under conditions of high salinity [33], this seemed unrelated to the observed growth phenotype of the ΔRnaC/S1022 mutant on M9 medium. In addition, two consistently predicted targets are involved in cell division (*racA* and *ftsW*), but we observed no specific cell-division abnormalities of the ΔRnaC/S1022 strain by live-imaging microscopy. Furthermore, the TCA cycle genes *citB* and *citZ* were predicted targets and tested by Western blot analysis, but no deregulation was observed. The last consistently predicted target of initial interest was the gene for the transition state regulator AbrB (Fig. 1D). Reviewing the literature on *abrB* pointed us to an interesting observation where a *spo0A* mutant was reported to display increased growth rates on media similar to our M9 medium [22]. Furthermore, it had been reported that AbrB has an additional role in modulating the expression of some genes during slow growth in suboptimal environments [34], which we argued could also be relevant to the M9S growth condition. Since *abrB* is a consistently predicted target of RnaC/S1022 (Fig. 1D), we checked whether the presence of this sRNA coincides with the presence of the *abrB* gene. Indeed, *abrB* is conserved in 53 out of 62 available *Bacillus* genomes, and RnaC/S1022 is present in 19 of these 53 genomes (Fig. 1A). In addition, we identified no genomes that contain RnaC/1022 but lack the *abrB* gene (Fig. 1A). Accordingly, we hypothesized that RnaC/S1022 might be a regulator of AbrB.

### AbrB levels are elevated in an RnaC/S1022 mutant

**Fig 2:**
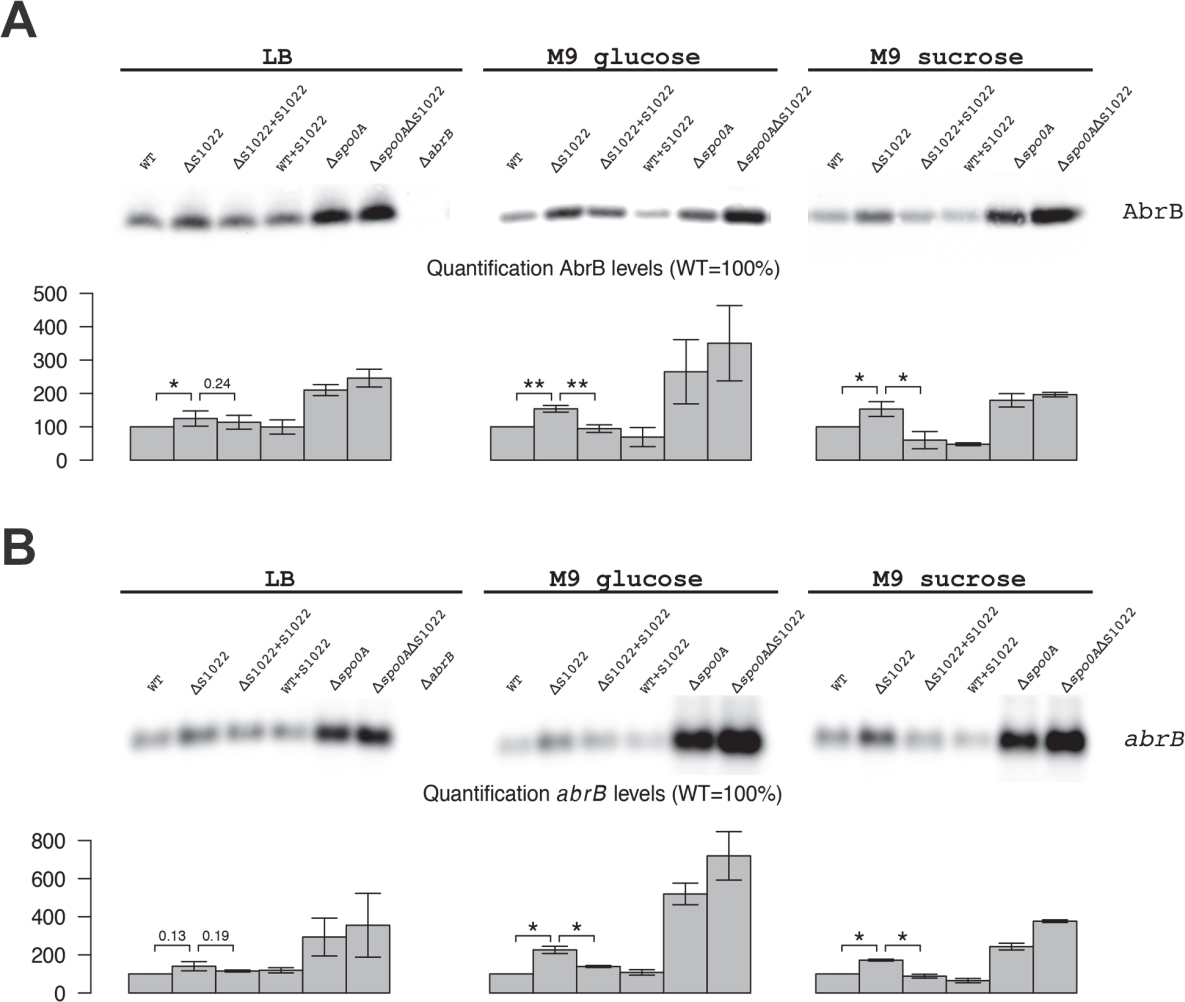
AbrB levels are dependent on the presence of RnaC/S1022. A) AbrB Western blot analysis. The position of AbrB is indicated. The bar diagrams show the relative AbrB levels, with the level in the parental strain (wt) set at 100%. All AbrB levels were corrected for the internal control protein BdbD. Error bars represent the standard deviation between triplicate experiments. The effect of RnaC/S1022 absence is most pronounced in cells grown on M9G and M9S, which corresponds to higher expression levels under these growth conditions. Statistical data analyis was performed with a one-sided Welch two-sample t-test (H_1_: AbrB/*abrB* levels in ΔRnaC/S1022 > than in the parental strain and the RnaC/S1022 complementation strain). The respective p-values are either indicated, or marked with asterisks (* p-value <0.05; ** p-value <0.01). B) *abrB* Northern blot analysis. Equal amounts of RNA were loaded in each lane. The bar diagrams show the relative *abrB* mRNA levels, with the level in the parental strain (wt) set at 100%. Quantifications are based on minimally two independent experiments. Error bars represent the standard deviation between experiments. Data was analyzed for significance as in A.

The combined clues from bioinformatics analyses and literature suggested that the growth phenotype of the ΔRnaC/S1022 mutant could relate to elevated AbrB levels. To test whether AbrB levels are indeed altered in this mutant, we performed Western blot and Northern blot analyses. This indeed revealed a strong trend towards higher AbrB protein and mRNA levels in the RnaC/S1022 mutant and for cells grown in M9G or M9S this effect was statistically significant (Fig. 2). Importantly, the growth phenotype as well as AbrB protein and mRNA levels returned to wild-type (wt) by ectopic expression of RnaC/S1022 under control of its native promoter from the *amyE* locus (Fig. 1C and 2). We also tested the effects of a Δ*spo0A* mutation by Western and Northern blot analyses. Interestingly, the combined deletion of RnaC/S1022 and *spoOA* seemed to lead to a further increase in the AbrB protein and mRNA levels compared to the already elevated levels in the *spo0A* mutant background. Lastly, we observed a three-fold reduced natural competence of the ΔRnaC/S1022 mutant, which is expected when the AbrB levels are elevated [35] (S3 Fig.).

To test whether the AbrB levels were directly dependent on RnaC/S1022 levels, we placed the RnaC/S1022 complementation cassette in the *amyE* locus of the parental strain and used Western and Northern blotting to measure AbrB protein and mRNA levels. These analyses showed a trend towards reduction of both the AbrB protein and mRNA levels in cells grown on M9G and M9S, which would be consistent with elevated RnaC/S1022 expression and increased *abrB* regulation (Fig. 2). Since the amount of AbrB was apparently correlated to the amount of RnaC/S1022, this suggested a stoichiometric relationship between these two molecules.

Before testing whether there could be a direct interaction between RnaC/S1022 and the *abrB* mRNA, we decided to investigate the fate of the *abrB* mRNA in the presence or absence of RnaC/S1022. For this purpose, we assayed the levels of the *abrB* mRNA at different time points after blocking transcription initiation with rifampicin in the RnaC/S1022 mutant strain and in the strain with two chromosomal copies of RnaC/S1022. This analysis showed that the *abrB* mRNA level decreased significantly faster in the presence of RnaC/S1022 than in its absence (S4 Fig.). In case of a direct interaction between RnaC/S1022 and the *abrB* mRNA, the observed difference could relate to an RnaC/S1022-triggered degradation of the *abrB* mRNA. Alternatively, this difference could be due to an RnaC/S1022-precluded protection of the *abrB* mRNA by elongating ribosomes [36].

### The RnaC/S1022 sRNA regulates AbrB by a direct sRNA-mRNA interaction

**Fig 3:**
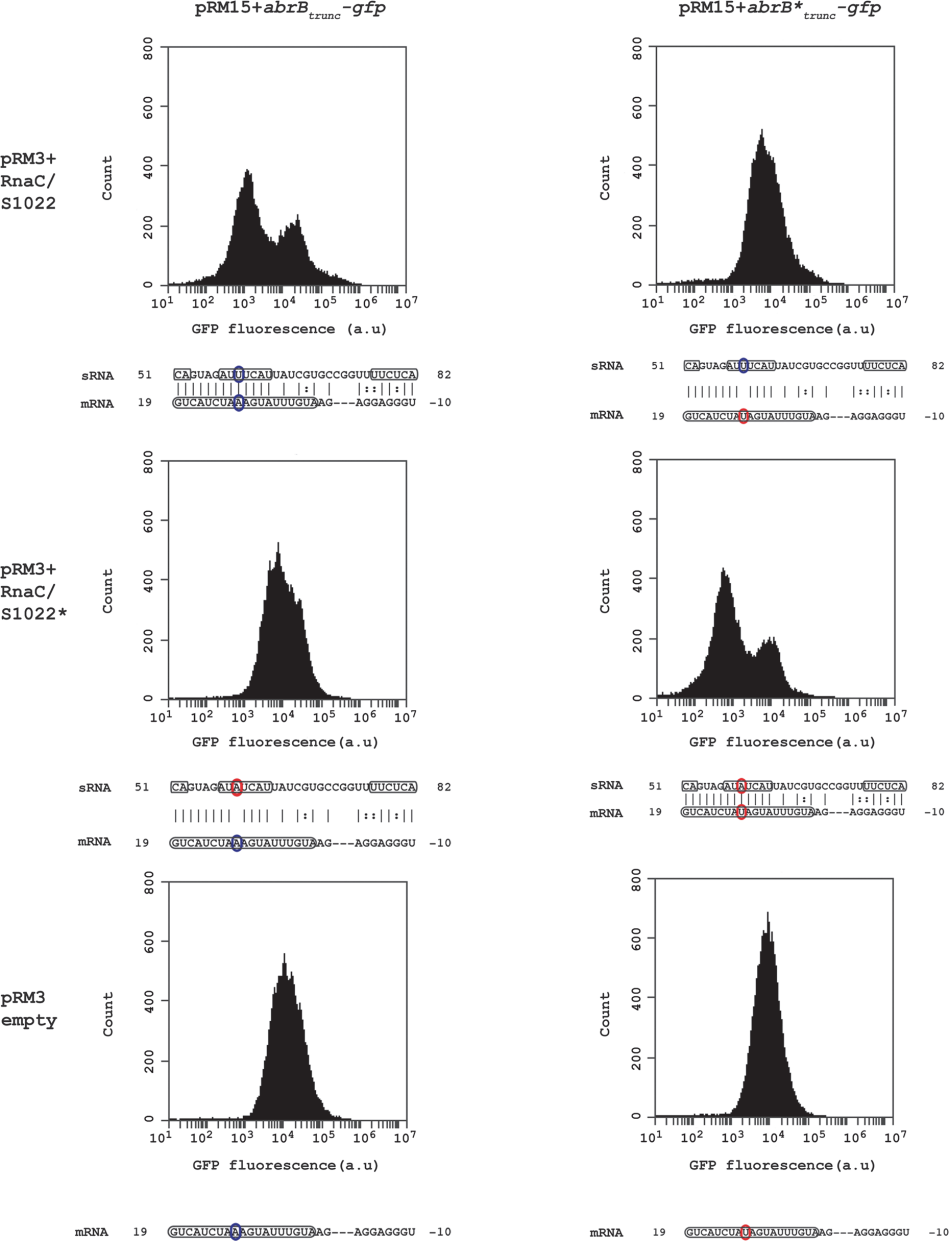
RnaC/S1022 regulates abrB by a direct mRNA-sRNA interaction. Wild-type and base pair-substituted (marked with asterix *) *abrB*_*trunc*_-*gfp* constructs were expressed from plasmid pRM15 and combined with one of three variants of the pRM3 plasmid (pRM3+RnaC/S1022, pRM3+RnaC/S1022*, pRM3 empty). All combinations of these constructs were assayed by FC and one representative histogram per combination is shown. Base-pairs high-lighted in identical color indicate the possibility for regulation due to base-pairing, while bases highlighted in two different colors indicate an inability for regulating due to the absence of base-pairing. Boxed regions in the *abrB* mRNA indicate the coding sequence, and boxed regions in the sRNA (RnaC/S1022) indicate predicted exposed bases in the structure shown in Fig. 1B. The base pair found to be essential for sRNA regulation corresponds to U59 in a predicted RnaC/S1022 loop region (RnaC/S1022* U59A) and the 11^th^ base in the *abrB-*encoding region (abrB*A11U).

The apparently stoichiometric relationship between AbrB and the sRNA RnaC/S1022 is suggestive of a direct sRNA—target interaction. The predicted interaction region in *B*. *subtilis* 168 spans a region from the RBS of *abrB* (-10) until 19 bp after the start of the *abrB* ORF of which the strongest consecutive stretch of predicted base-pair interactions are present from +7 bp till +19 bp (left top panel in Fig. 3). In addition, only this region within the *abrB*-encoding sequence is part of the conserved predicted interaction region in *B*. *atrophaeus* and *B*. *subtilis spizinenzii* (Fig. 1D). It has been reported that loop-exposed bases of sRNAs are more often responsible for regulation than bases in stems [37]. Two predicted loop regions of RnaC/S1022 are complementary with the predicted *abrB* interaction region (one of two basepairs and one of seven basepairs; bases 51–52 and 57–63 in Fig. 1B and 3). We therefore decided to introduce a point-mutation by a U to A substitution in the predicted 7-bp loop of RnaC/S1022 encoded by plasmid pRM3 and a compensatory mutation in a plasmid pRM15-borne truncated *abrB-gfp* reporter construct (*abrB*_*trunc*_-*gfp*). Strains containing different combinations of the respective plasmids were grown on M9G and assayed by Flow Cytometry (FC) in the exponential growth phase. Cells containing one of the *abrB*_*trunc*_-*gfp* constructs in combination with the empty pRM3 plasmid displayed a unimodal distribution in GFP levels (Fig. 3, lower panels). However, when the wt *abrB*_*trunc*_-*gfp* was assayed in combination with the wt RnaC/S1022, a bimodal distribution in AbrB_trunc_-GFP levels was observed, including a new peak of lowered fluorescence intensity (Fig. 3, top left). Interestingly, a unimodal fluorescence distribution was found when the wt *abrB*_*trunc*_-*gfp* construct was combined with point-mutated RnaC/S1022* (Fig. 3, middle left) or the mutated abrB*_trunc_-*gfp* with the wt RnaC/S1022 (Fig. 3, top right). In the case of the point-mutated abrB*-*gfp* construct, however, a bimodal fluorescence distribution was only observed when this construct was combined with the mutated RnaC/S1022* (Fig. 3, middle right). This implies that a direct mRNA-sRNA interaction takes place between *abrB* and RnaC/S1022.

### RnaC/S1022 sRNA is condition-dependently expressed

**Fig 4:**
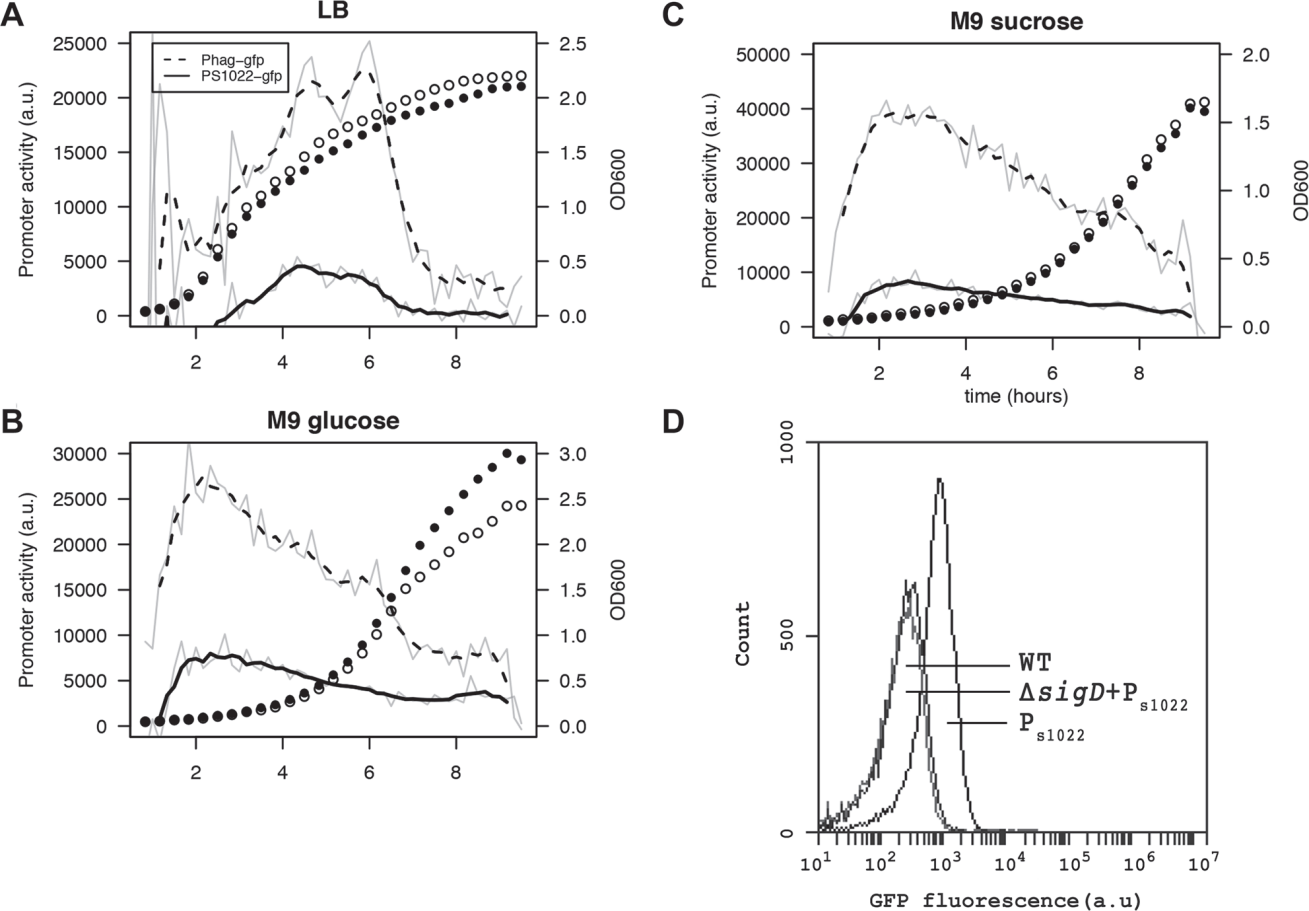
RnaC/S1022 is condition-dependently expressed. Promoter activity of the P_RnaC/S1022_-*gfp* and P_*hag*_-*gfp* fusions in cells grown on LB (A), M9G (B), and M9S (C). Promoter activities were computed by subtraction of GFP level from the previous time-point as described by Botella et al. [38]. The experiment was performed three times in triplicate. Average data from triplicate measurements of one representative experiment are shown. The grey line indicates mean data and the black line a smoothed version of this mean. One in two time-points were plotted for the growth curve as open circles for the P_*hag*_-*gfp* strain and closed circles for the P_RnaC/S1022_-*gfp* strain. D) Representative FC results for P_RnaC/S1022_-*gfp* expression in mid-exponentially growing cells with or without a *sigD* deletion. The cells were grown in M9G. Wt, *B*. *subtilis* 168 not expressing GFP.

Studying the condition-dependency of sRNA expression can give clues to its function and targets. To obtain high-resolution expression profiles, we constructed an integrative RnaC/S1022 promoter-*gfp* fusion [38]. As expected, the presence of this P_RnaC/S1022_-*gfp* fusion caused GFP fluorescence in wild-type cells, but not in cells with a *sigD* mutation (Fig. 4D). Next, a live cell array approach was used to compare the P_RnaC/S1022_-*gfp* activity with that of another SigD-dependent promoter, P_*hag*_, which drives flagellin expression. These promoter fusion strains revealed that the expression of *hag* was consistently ∼4 fold higher than that of RnaC/S1022 (Fig. 4), which is in agreement with previously published expression data [9]. On LB medium, the expression of both RnaC/S1022 and *hag* peaked in the late exponential and transition phase, while on both tested minimal media the peak in expression occurred in early exponential phase (Fig. 4). This higher RnaC/S1022 expression level in the exponential phase on M9 relative to that in LB is in concordance with the stronger effect of ΔRnaC/S1022 on AbrB levels, as indicated by the Northern and Western blot analyses.

### The RnaC/S1022 sRNA modulates protein expression noise of AbrB-GFP

Experimental methods that measure average protein levels in a population obscure possible cell-to-cell variation. To further study the cell-to-cell variation of AbrB-GFP in the exponential growth phase (as observed in Fig. 3), we therefore employed a full-length translational *abrB*-*gfp*mut3 fusion that was integrated into the chromosome via single cross-over (Campbell-type) recombination. Specifically, this integration resulted in a duplication of *abrB* where one full-length copy of *abrB* was expressed from its own promoter and fused in-frame to *gfp*, while the downstream *abrB* copy was truncated lacking the start codon required for translation [39]. In this AbrB-GFP strain all AbrB monomers have a C-terminally attached GFP molecule. While AbrB-GFP still localized to the nucleoid (S5 Fig.), this AbrB-GFP strain displayed a somewhat reduced growth rate on media where AbrB is required for rapid growth. Since the translational *abrB*-*gfp* fusion is chromosomally integrated at the *abrB* locus, this system is insensitive to fluctuations in noise levels by plasmid copy number variation and its chromosomal location in the division cycle.

We first used the AbrB-GFP fusion to test whether *B*. *subtilis* Hfq might have an effect of the RnaC/1022-*abrB* interaction. Consistent with previous studies on other sRNA targets of *B*. *subtilis* [5–7], the direct RnaC/S1022-*abrB* regulation was found to be independent of Hfq since comparable FC profiles for AbrB-GFP expression were obtained for the parental strain and the *hfq* deletion mutant (S6 Fig.).

**Fig 5:**
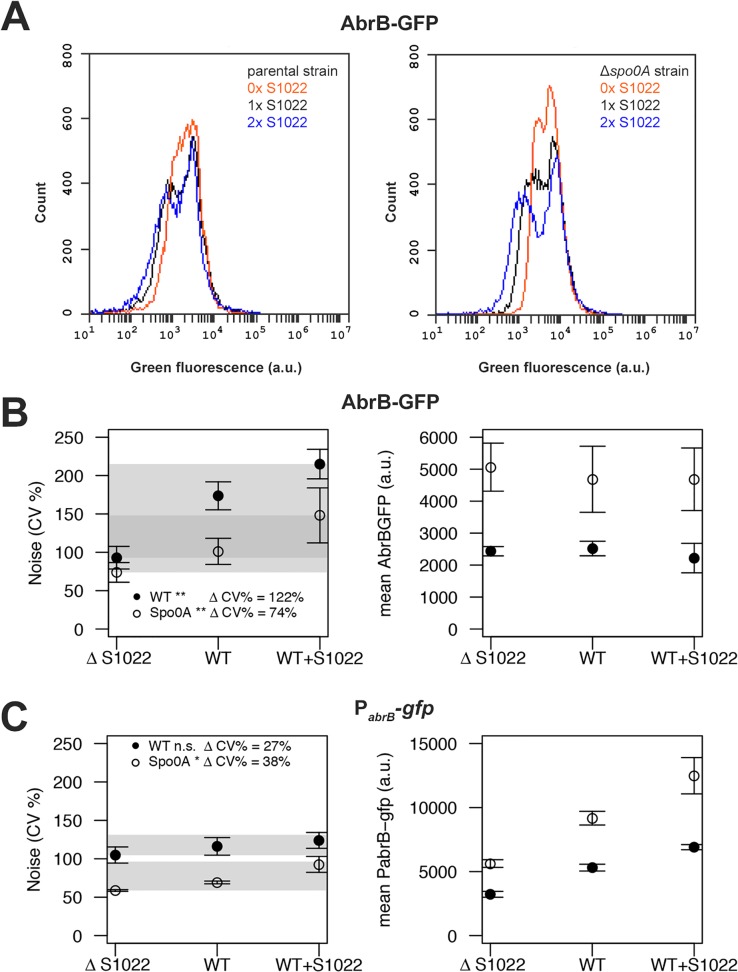
RnaC/S1022 induces protein expression noise of AbrB-GFP. A) Representative FC data for the translational AbrB-GFP fusion expressed from a single copy of the respective gene fusion integrated into the *B*. *subtilis* chromosome. All strains were grown on M9G. The left panel shows histograms for, from left to right, the AbrB-GFP-producing strain with two chromosomal RnaC/S1022 copies, the AbrB-GFP-producing parental strain with one chromosomal RnaC/S1022 copy, and the AbrB-GFP-producing strain with the ΔRnaC/S1022 mutation. The right panel shows data for the same strains with an additional Δ*spo0A* mutation. Please note the increase in the width of the distribution with increasing RnaC/S1022 gene dosage. B) Quantification of AbrB-GFP noise (left panel) and mean expression data (right panel) from three independent experiments with cells grown on M9G. Shaded areas indicate the noise increase (ΔCV%) from 0 to 2 sRNA copies in the *spo0A*-proficient background (wt) and the Δ*spo0A* mutant background. Statistical significance of the comparisons of data obtained for *spo0A*-proficient or -deficient strains containing 0 to 2 sRNA copies are indicated with asterisks in the legend (* p-value <0.05; ** p-value <0.01; ANOVA with Tukey HSD test). Error bars represent the standard deviation. C) Quantification of P_*abrB*_-*gfp* noise (left panel) and mean P_*abrB*_-*gfp* activity data (right panel) from three independent experiments with cells grown on M9G. Statistical significance of the comparisons of data obtained for *spo0A*-proficient or -deficient strains containing 0 to 2 sRNA copies are indicated with asterisks in the legend (* p-value <0.05; n.s. means not significant; ANOVA with Tukey HSD test). Error bars represent the standard deviation.

Next, we analyzed all AbrB-GFP strains by FC in the exponential phase on both LB and M9G. Noise measurements were not performed on M9S because of the strong growth difference between the parental and ΔRnaC/S1022 strains on this medium (Fig. 1C). We observed that the difference between cells expressing AbrB-GFP at the highest level and those at the lowest level was large (Fig. 5A). This means that AbrB-GFP is expressed with high noise (quantified as the coefficient of variation; CV%). Interestingly, we observed lower AbrB-GFP noise in strains lacking RnaC/S1022 and, crucially, the presence of an additional genomic RnaC/S1022 copy further increased AbrB-GFP noise. Remarkably, increased RnaC/S1022 levels only reduced the minimal expression level of the distribution while not affecting the maximum AbrB-GFP expression level (Fig. 5A), which is consistent with the data presented in Fig. 3. There was a statistically significant positive linear correlation between RnaC/S1022 levels (0, 1 or 2 genomic copies) and AbrB-GFP noise (on LB for pooled data points from Δ*spo0A* and parental backgrounds R^2^ 0.48, P-value <0.001, and M9G R^2^ 0.43, P-value <0.001) (Fig. 5B). Direct statistical comparisons between AbrB-GFP noise levels at different sRNA levels also revealed significant changes (Fig. 5). The noise increase therefore seems correlated to the level of RnaC/S1022. Notably, this relation was also observed for noise measurements in a *spo0A* deletion background, even though the mean AbrB-GFP expression was between 1.37 and 2.32 fold (for LB and M9G respectively, μ = 1.79) higher in Δ*spo0A* strains. This suggests that RnaC/S1022 has a specific role in noise modulation of AbrB-GFP.

### RnaC/S1022 has no indirect effect on the abrB promoter and is expressed homogeneously

After observing that RnaC/S1022 specifically increases AbrB-GFP expression noise, we aimed to elucidate the origin of this AbrB-GFP noise. Three possibilities for noise generation by an sRNA are conceivable. Firstly RnaC/S1022 could have an additional indirect effect on *abrB* expression, leading to noisy expression from the *abrB* promoter and subsequent propagation of this noise to the AbrB protein level. Secondly, RnaC/S1022 may itself be expressed either in bimodal fashion or with high noise. The third possibility would be an AbrB-dependent repression of the RnaC/S1022 promoter and subsequent repression of AbrB protein levels by RnaC/S1022. This double negative repression would correspond to positive feedback on the AbrB protein level, and positive feedback is a known source of expression heterogeneity [40].

To study the distribution of the *abrB* promoter, we integrated the pBaSysBioII plasmid [38] directly behind the Spo0A binding site in the promoter region of *abrB* [41], resulting in a single-copy promoter fusion at the native genomic locus (P_*abrB*_; -41bp of the *abrB* start codon). This location was selected to include the effect of AbrB autorepression and Spo0A(-P) repression, while excluding RnaC/S1022 regulation. We observed no bimodal or particularly noisy expression of this *abrB* promoter fusion, showing that transcription from the *abrB* promoter is homogeneous in the exponential phase (Fig. 5C). Of note, bimodal or noisy expression of P_*abrB*_ would have been surprising since transcription of *abrB* is autorepressed and it is generally found that this NAR reduces the noise of promoter expression [42, 43]. Interestingly, the expression from the *abrB* promoter rises with increasing levels of RnaC/S1022. This observation can be explained by AbrB autorepression and noise. There are more cells with low AbrB levels when the levels of RnaC/S1022 are increased. On average, this will lead to lowered repression of the *abrB* promoter, leading to a higher level of expression (but not more noise) from the *abrB* promoter (Fig. 5C). This higher expression from the *abrB* promoter is apparently compensated for at the protein level by the elevated regulation of RnaC/S1022 (Fig. 2 and 5B). Since we observed only a slight increase in *abrB* promoter noise specific to RnaC/S1022 (Fig. 5C), the hypothesis that AbrB-GFP noise promotion originates from an additional effect of RnaC/S1022 on the *abrB* promoter can be rejected.

A second possibility of noise promotion by RnaC/S1022 is that it is itself expressed with large noise similar to the SigD-dependent *hag* gene [44]. In this case, large cell-to-cell variation in sRNA levels would only lead to regulation in cells that have above-threshold sRNA levels, and this could generate the variation in AbrB-GFP levels. We tested this at the promoter level by FC analysis of the integrative RnaC/S1022 promoter-*gfp* fusion (P_RnaC/S1022_; Fig. 4) and found this promoter fusion to be homogenously expressed with a tight distribution of GFP levels (CV% of 64% for the M9G condition; Fig. 4D). Furthermore, we argued that the relatively low expression of P_RnaC/S1022_ could result in threshold-level regulation where the sRNA is only involved in regulating *abrB* in cells with above-threshold levels of RnaC/S1022. However, this is not consistent with the observation of further increased noise levels in cells with two genomic copies of RnaC/S1022 (Fig. 5B). We therefore consider the possibility of AbrB noise promotion via heterogeneous expression of RnaC/S1022 unlikely. It cannot be excluded, however, that variation in the levels of RnaC/S1022 might be introduced further downstream, for instance via mRNA degradation, or via regulation by a dedicated RNA chaperone.

The third option would be a double-negative feedback loop consisting of sRNA repression of AbrB levels and AbrB repression of sRNA levels, which would ultimately lead to an increase in AbrB protein expression noise. This would thus depend on repression of the RnaC/S1022 promoter by AbrB, in addition to the confirmed negative regulation of *abrB* by RnaC/S1022. Together this would lead to a decrease in AbrB protein levels in cells that start with below-threshold AbrB levels. First of all, we found no indication for AbrB binding sites in the region upstream of RnaC/S1022 in the dataset of Chumsakul et al., where the binding sites of AbrB were mapped genome-wide [24]. To test whether the RnaC/S1022 promoter is indeed not directly controlled by AbrB, or possibly under indirect control of AbrB, we deleted the *abrB* gene from the above-mentioned P_RnaC/S1022_ promoter fusion strain. As expected, the RnaC/S1022 promoter activity levels were not detectably affected by the *abrB* deletion (S7 Fig.), showing that it is unlikely that there is a negative feedback loop consisting of AbrB-dependent RnaC/S1022 repression and RnaC/S1022-dependent *abrB* repression.

### sRNA-induced protein expression noise is consistent with mathematical models of sRNA regulation

**Fig 6:**
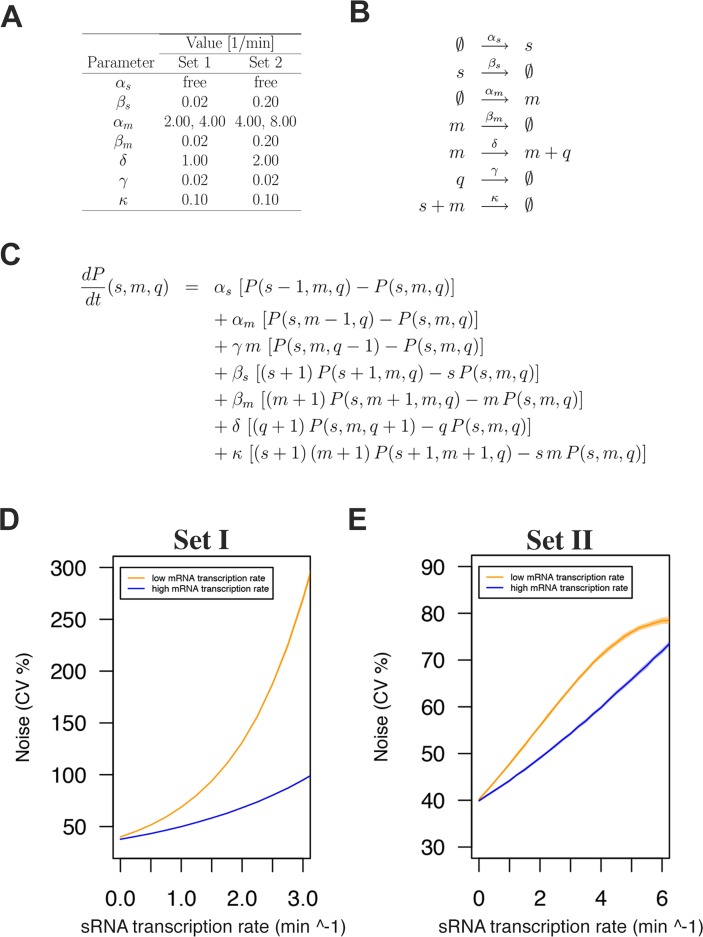
sRNA regulation increases protein expression noise in a stochastic simulation model. A) Noise-generating dynamics of sRNA regulation were simulated in a stochastic simulation model for two sets of parameters with two mRNA transcription rates and the sRNA transcription rate as a free parameter. B) Reactions considered in the model. C) Master equation used for the model. D) Modeling outcome for parameter Set I. E) Modeling outcome for parameter Set II. Note that modeling with both parameter sets predicts increased protein expression noise with increased sRNA transcription rates. In both cases this effect is buffered by a higher mRNA transcription rate as was observed for the Δ*spo0A* mutant background (see Fig. 5B).

Since the experimental data presented above pointed to a direct role of the RnaC/S1022 sRNA in AbrB protein noise promotion, we wondered whether this possibility is consistent with mathematical models of sRNA regulation. To verify this, we considered a simple model of RNA regulation with two independently transcribed RNA species (sRNA and mRNA) [45–47]. In this model, these molecules are synthesized with constant transcription rates α_s_ and α_m_, respectively. Translation of mRNA into protein *Q*, and the degradation of sRNA, mRNA, and protein molecules were modeled as linear processes that occur with rates δ. β_s_, β_m_, and γ, respectively. The sRNA-mRNA duplex formation was assumed to be an irreversible second-order process that occurs with a rate κ. In the model, molecules in the sRNA-mRNA duplex were removed from the dynamical system. A summary of all reactions and the master equation used in the model can be found in Fig. 6.

We first implemented model parameters used in an earlier sRNA modeling study by Jia et al. [47] (Set I; Fig. 6A and D). Of note, these parameters were essentially the same as those of Levine et al. [45]. In all cases the sRNA transcription rate (α_s_) was a free variable to capture the effect of 0, 1, or 2 genomic copies of RnaC/S1022. In addition, for each set of parameters we included two possible α_m_ values to model the effect in the Spo0A deletion strain where the *abrB* transcription rate (α_m_) is approximately two-fold higher than in the parental strain (as determined with P_abrB_-*gfp*). Varying extrinsic noise in the *abrB* transcription rate had no effect on the general modeling outcome (S8 Fig.) and the intermediate α_m_ CV% level of 40% was selected for plots in the main text. After running the model with parameters from Set I, we observed that model-predicted protein noise strongly increased with increasing α_s_. This trend of increasing protein noise with increasing sRNA transcription rates was similar to what we observed for the genomic AbrB-GFP fusion (Fig. 5A and B). Importantly, doubling α_m_ (two-fold higher mRNA transcription rate) resulted in a more gradual noise increase with increasing α_s_, just as was observed in the Δ*spo0A* mutant with the AbrB-GFP fusion (Fig. 5).

We next sought to determine the effect of changing modeling parameters on the modeling outcome, because the selected mRNA half-life of ∼35 min (β_m_ 0.02) in parameter Set I would only be relevant for a subset of mRNA molecules as shown experimentally by Hambraeus et al. [48] (with a relation between these of mean lifetime from Fig. 6A * ln 2 = half-life). We therefore constructed a second set of modeling parameters (Set II), which gave the mRNA and sRNA species a half-life of ∼3.5 min (β_m_ and β_s_ 0.20) while keeping protein half-life at ∼35 min. In addition, α_m_ was increased from 2 transcripts per minute to 4 per minute, and δ was doubled to 2 synthesized proteins per minute. Although the maximum noise level from these Set II simulations was markedly different, it again clearly showed the trend of increasing protein noise with increasing sRNA transcription rates. We can therefore conclude that the modeling results robustly support the idea that sRNA regulation can generate noise at the protein level. This noise would be induced locally at the level of mRNA degradation or translation initiation, and the corresponding fluctuations would subsequently be propagated to the protein level. Recently, the theoretical background of this concept was also reported by Jost et al., who stated that such behavior is especially expected when the levels of the srRNA and the mRNA are approximately equal [49]. Altogether, our experimental data and the modeling approach are consistent with the view that RnaC/S1022 is an intrinsic noise generator for AbrB-GFP at the post-transcriptional level.

### RnaC/S1022-induced AbrB expression noise generates diversity in growth speeds in the exponential phase

**Fig 7:**
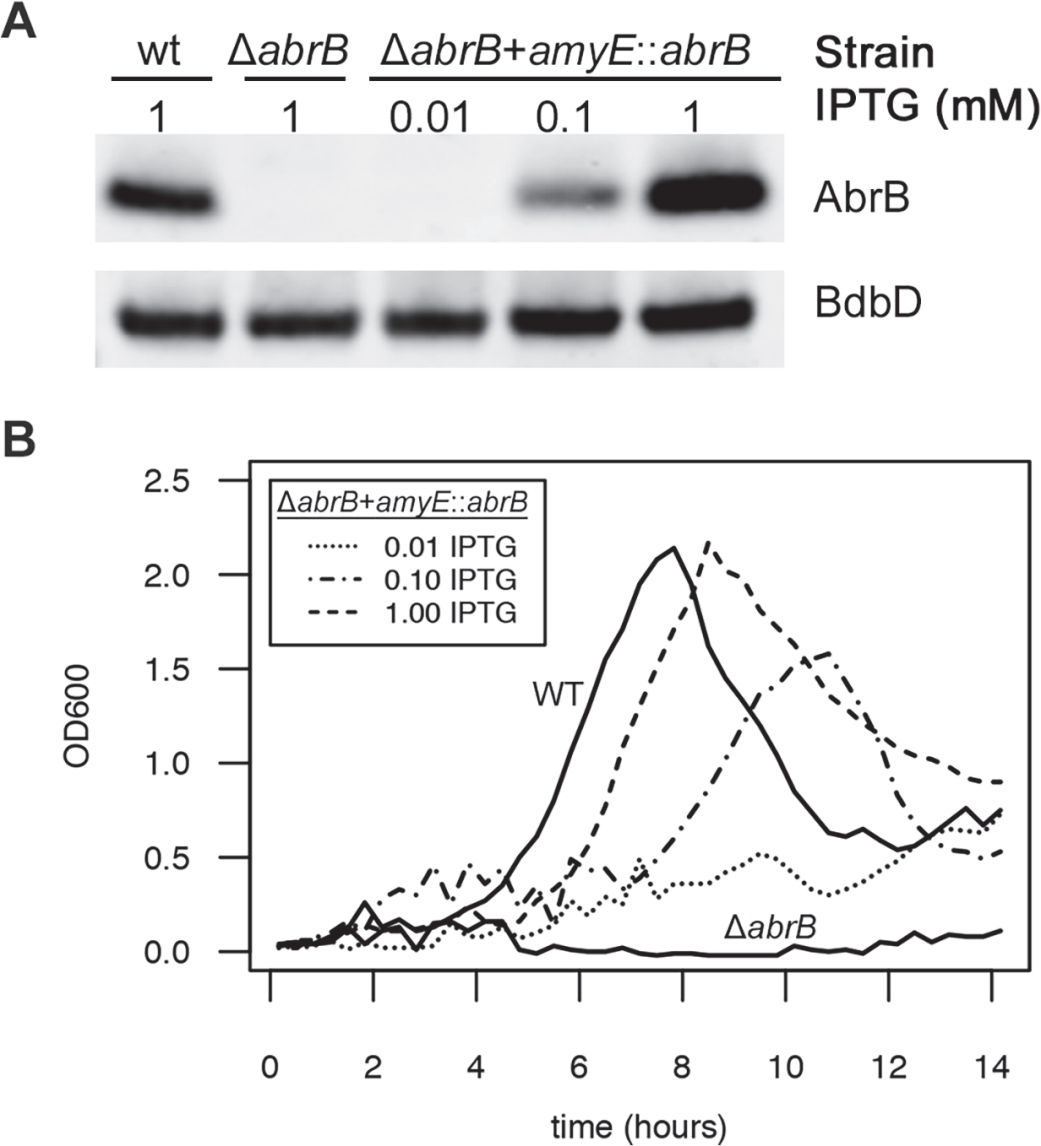
AbrB levels determine the growth rate and growth yield on M9 minimal medium. A) Representative Western blot data for AbrB production by the indicated strains grown on LB medium as a test for IPTG-dependent AbrB production from the *amyE*::*abrB* construct. At the lowest level of IPTG induction (0.01 mM), the AbrB production remained below the detection level. BdbD was used as a loading control. AbrB and BdbD were visualized with specific antibodies. B) Growth profiles on M9G determined for the strains from panel A. The *abrB* mutant is unable to grow on this medium, but its growth defect is rescued by IPTG-induced AbrB expression from the *amyE*::*abrB* construct. Averages of triplicate measurements from one representative 96-well plate experiment are shown.

After defining the experimental and theoretical framework for noise promotion by the RnaC/S1022 sRNA, we wondered what the physiological relevance of this regulation might be. Since we and others (Fig. 1A; [22]) have reported an effect of AbrB levels on the growth of *B*. *subtilis*, a growth-related function seemed obvious. We therefore tested whether AbrB levels are a direct determinant of growth rate and yield under the relevant conditions. To do this, we placed the *abrB* gene under control of an isopropyl ß-D-1-thiogalactopyranoside (IPTG)-inducible promoter in the *amyE* locus using plasmid pDR111 [50], and subsequently deleted the *abrB* gene from its native locus in this strain. We first verified the IPTG-dependent expression of AbrB from this construct by growing the parental strain, the Δ*abrB* strain, and the Δ*abrB amyE*::*abrB* strain on LB medium and, in the case of the Δ*abrB amyE*::*abrB* strain, the medium was supplemented with increasing IPTG concentrations. Subsequently, AbrB production was assessed by Western blot analysis (Fig. 7A), which showed that AbrB production in the Δ*abrB amyE*::*abrB* strain was indeed IPTG-dependent. Notably, the *abrB* mutant strain displays a growth phenotype on LB medium, but this is only apparent in the late exponential growth phase [51]. To analyze the effect of differing AbrB levels on growth under conditions that are more relevant for the RnaC/S1022—*abrB* interaction, we grew the same strains on M9G, which was supplemented with differing amounts of IPTG for the Δ*abrB amyE*::*abrB* strain. As shown in Fig. 7B, the *abrB* deletion mutant did not grow in this medium. Importantly however, IPTG-induced expression of *abrB* in this mutant repaired the growth phenotype in a dose-dependent manner. This shows that the AbrB levels determine the growth rate and yield when cells are cultured on M9G.

**Fig 8:**
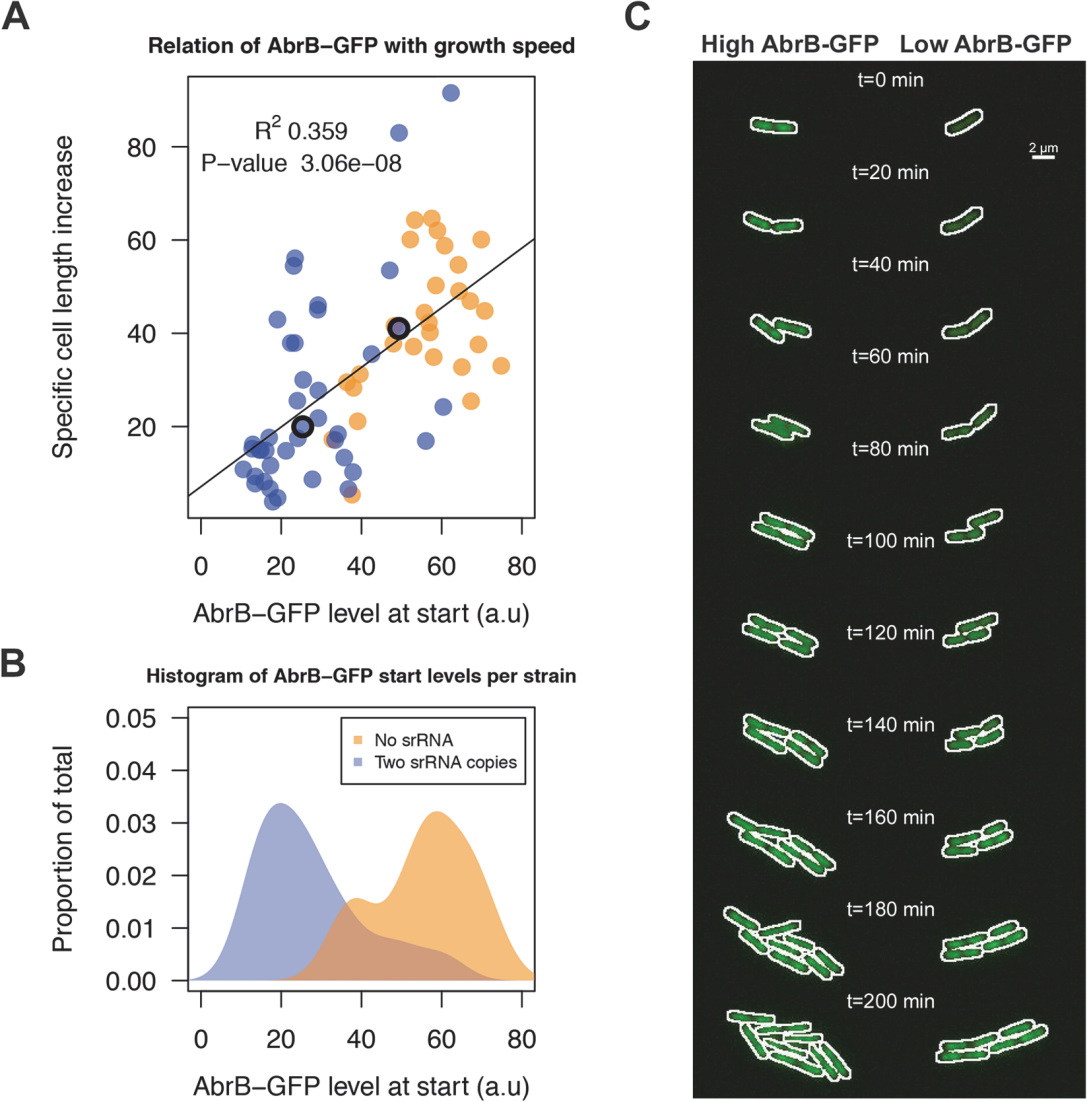
RnaC/S1022-induced variation in AbrB-GFP levels leads to heterogeneity in growth rates. A) Tracing of growth and AbrB-GFP levels of 71 individual micro-colonies from Δ*spo0A* AbrB-GFP strains with either zero or two chromosomal copies of RnaC/S1022. Data originates from three independent experiments. Cell growth is expressed as the cell length (Feret’s diameter) increase per hour as determined in the first 20 min after spotting of the cells onto agarose slides. The plotted AbrB-GFP level is the average of fluorescence in the first and second picture. B) Distribution of AbrB-GFP start levels for both strains. Note that two genomic RnaC/S1022 copies lead to a wider distribution of AbrB-GFP levels. C) Montage of the two adjacent dividing cells from the S1 Movie. The white outline marks the contours of the cell. The positions of these cells in panel A are marked with an **O**. Individual cells were cropped for illustration purposes only.

We next aimed to unravel the effect of sRNA-induced AbrB heterogeneity on growth. This requires the tracking of cells with low and high AbrB-GFP levels over time. To do this, we performed a live imaging experiment with the Δ*spo0A* AbrB-GFP strain either containing zero sRNA copies due to the ΔRnaC/S1022 mutation, or two genomic copies due to the insertion of an additional RnaC/S1022 copy in *amyE*. The Δ*spo0A* background was used to elevate AbrB-GFP levels and thereby to facilitate fluorescence measurements. Cells were pre-cultured in M9G as was done for the FC measurements and applied to agarose pads (at OD_600_ ∼0.15) essentially as was described by Piersma et al. [52]. From these experiments, and consistent with FC data in Fig. 5, it was apparent that there was a larger variation in AbrB-GFP levels in the strain with two genomic RnaC/S1022 copies, compared to the strain lacking RnaC/S1022 (Fig. 8B; S1 Movie). In addition, this variation in AbrB-GFP levels was correlated to the variation in growth rates (quantified as the specific cell length increase) observed during the first 20 min of each live imaging run (Fig. 8A). We excluded the possibility that this growth rate difference was dependent on the position on, or quality of the slide. Instead, it was solely linked to the cellular level of AbrB-GFP (Fig. 8C; S1 Movie).

Notably, in the two example cells from Fig. 8C (S1 Movie) AbrB-GFP levels gradually increase in the cell with a low start level (i.e. high level of sRNA repression), which would be consistent with a gradual reduction in RnaC/S1022 expression on this solid agarose medium (S9 Fig.). However, our experimental setting determines the effect of AbrB-GFP on growth before this reduction in RnaC/S1022 becomes relevant (e.g. the first 5 pictures, or 20 min) (Fig. 8A). Beyond this, the increase in AbrB-GFP levels observed later (>150 min) in the live imaging experiment seems coupled to a concomitant increase in growth rate (S9 Fig.). This is again consistent with the positive correlation of AbrB-GFP levels with growth rate. Interestingly, while we observed a few cells switching their AbrB-GFP expression state from high to low, the AbrB-GFP levels were generally stable throughout a cell’s lineage. Combined, these analyses show that the RnaC/S1022-induced heterogeneity in the AbrB-GFP expression levels generates diversity in growth rates within the exponential phase of growth.

## Discussion

In this study we show that *B*. *subtilis* employs the RnaC/S1022 sRNA to post-transcriptionally regulate AbrB and that this regulation results in increased heterogeneity in growth rates during the exponential phase of growth. RnaC/S1022 is the third sRNA in *B*. *subtilis* for which a direct target has been reported and this study reveals the value of evolutionary target predictions to identify true sRNA targets for this species.

The observed growth rate heterogeneity induced by RnaC/S1022 is conceivably of physiological relevance since slowly growing bacterial cells are generally less susceptible to antibiotics and other environmental insults than fast growing cells [53–55]. Specifically, it was noted for *hip* strains of *E*. *coli* that slowly growing cells within a population will develop into persister cells when challenged with ampicillin [17]. Notably, in this system, the initial heterogeneity in growth rates was reported to be dependent on the HipAB toxin-antitoxin module [56]. Analogously, it is conceivable that a *B*. *subtilis* toxin-antitoxin module under negative AbrB control could be responsible for the heterogeneity observed in the present study. Another perhaps more likely possibility is that low AbrB levels cause the premature activation of transition- or stationary phase genes, thereby slowing down growth and causing premature stationary phase entry. AbrB has also been implicated in the activation of some genes when CCR is relieved [22, 23], and this could be related to the stronger growth phenotype observed on M9S compared to M9G. However, the AbrB level also determines growth rates on M9G (this study; [22]), when CCR is active and AbrB is not known to have an activating role [22].

The initially observed growth phenotype of the ΔRnaC/S1022 mutant can be explained by the present observation that AbrB is an important determinant for growth on M9 medium, and that RnaC/S1022 regulation of AbrB is specifically linked to increasing AbrB noise. Specifically, the absence of RnaC/S1022 will reduce the number of cells expressing AbrB at a low level. Growth of the ΔRnaC/S1022 population will therefore be more homogeneous and, when inspected as an average, the population will enter stationary phase later than the parental strain. Beyond the mechanism of AbrB-mediated growth regulation, we show that noisy regulation of a growth regulator can also cause heterogeneity in growth rates. This suggests that the AbrB noise level has been fine-tuned in evolution, possibly as a bet-hedging strategy to deal with environmental insults.

Two other questions addressed by this study are the origin of AbrB expression noise, and the likely reason why this noise is generated at the post-transcriptional level. The origin of AbrB expression noise via triggering of *abrB* mRNA degradation and/or inhibition of *abrB* translation fits the definition of an intrinsic noise source where the absence of RnaC/S1022 reduces the number of sources for intrinsic noise by one, and therefore results in lower protein expression noise. This specific noise-generating capacity of sRNA regulation might be due to the specific kinetics of the RnaC/S1022- *abrB* mRNA interaction. It is currently unclear whether this feature of sRNA-mediated regulation can be extended to other sRNA-mRNA pairs. Specifically, subtle consequences of sRNA regulation, such as noise generation, may have been overlooked in previous studies due to the use of plasmid-encoded translational fusions with fluorescent proteins expressed from strong non-native promoters as reporters. We therefore expressed all RnaC/S1022 and AbrB-GFP constructs from their native genomic location, from their native promoters, and assayed the effects in the relevant growth phase.

NAR of AbrB seems to be the answer to the second question why noise is generated post-transcriptionally and not at the promoter level. AbrB’s NAR is important for its functioning in the stationary phase sporulation network [26, 27] and is therefore likely a constraint for evolutionary optimization of AbrB expression in the exponential phase, which is the growth phase addressed in this study. In turn, NAR is a clear constraint on noise generation since it is generally believed to dampen noise [42, 43]. Consistent with this view, we observed only a slight increase in P_*abrB*_ promoter noise upon increasing AbrB protein noise, suggesting that AbrB NAR is responsible for minimizing promoter noise. Besides reducing noise, NAR has been implicated in decreasing the response time of a genetic circuit, linearizing the dose response of an inducer, and increasing the input dynamic range of a transcriptional circuit [19]. Individually, and in combination, these mechanistic aspects of NAR could explain why NAR is such a widespread phenomenon in transcriptional regulation. Besides this, the idea that AbrB and AbrB NAR are more widely conserved than RnaC/S1022 would be in line with the idea that AbrB expression in *B*. *subtilis* 168 has become fine-tuned by an additional regulator, which has evolved later in time. Lastly, on a more general note, the inconsistency between the *abrB* promoter and AbrB protein noise measurements make it clear that it is premature to draw conclusions about homogeneity or heterogeneity of protein expression when only data is gathered at the promoter level, especially for genes under a NAR regime.

In conclusion, we have identified a novel direct sRNA target in the important *B*. *subtilis* transcriptional regulator *abrB*. Specifically, we provide functionally and physiologically relevant explanations for the evolution of the noise-generation aspects of this regulation in generating heterogeneity in growth rates. This noise is induced at the post-transcriptional level due to AbrB NAR. Based on our present observations, we hypothesize that the resulting subpopulations of fast- and slow-growing *B*. *subtilis* cells reflect a bet-hedging strategy for enhanced survival of unfavorable conditions.

## Materials and Methods

### Bacterial strain construction

*E*. *coli* and *B*. *subtilis* strains and plasmids used in this study are listed in S2 Table and oligonucleotides in S3 Table. *E*. *coli* TG1 was used for all cloning procedures. All *B*. *subtilis* strains were based on the *trpC2*-proficient parental strain 168 [1]. *B*. *subtilis* transformations were performed as described previously [57]. The isogenic RnaC/S1022 mutant was constructed according to the method described by Tanaka et al. [58]. pRMC was derived from pXTC [59] by Circular Polymerase Extension Cloning (CPEC) [60] with primers ORM0054 and ORM0055 using pXTC as PCR template and ORM0056 to circularize this PCR fragment in the final CPEC reaction. In this manner, the xylose-inducible promoter of pXTC was replaced with the *Asc*I Ligation Independent Cloning (LIC; [61]) site from pMUTIN-GFP [39]. As a consequence, pRMC carries a cassette that can be integrated into the *amyE* locus via double cross-over recombination, allowing ectopic expression of genes in single copy from their native promoter. RnaC/S1022 was cloned in pRMC under control of its native promoter as identified by Schmalisch et al. [28], and the subsequent integration of RnaC/S1022 into the *amyE* locus via double cross-over recombination was confirmed by verifying the absence of α-amylase activity on starch plates. The LIC plasmid pRM3+P_wt_ RnaC/S1022, which is a derivative of plasmid pHB201 [51], was used to express RnaC/S1022 under control of its native promoter. For IPTG-inducible expression of *abrB*, the *abrB* gene was cloned into pDR111 [50], and subsequently placed in the *amyE* locus via homologous recombination. Deletion alleles were introduced into this and other strains by transformation with chromosomal DNA containing the respective mutations. The RnaC/S1022, *hag* and *abrB* promoter *gfp* fusions were constructed at the native chromosomal locus by single cross-over integration of the pBaSysBioII plasmid [38]. A minimum of three clones were checked to exclude possible multi-copy integration of the plasmid.

### Media and growth conditions

Lysogeny Broth (LB) consisted of 1% tryptone, 0.5% yeast extract and 1% NaCl, pH 7.4. M9 medium supplemented with either 0.3% glucose (M9G) or 0.3% sucrose (M9S) was freshly prepared from separate stock solutions on the day of the experiment as previously described [9]. For live cell imaging experiments, the M9 medium was filtered through a 0.2 μm Whatman filter (GE Healthcare). Strains were grown with vigorous agitation at 37°C in either Luria LB or M9 medium using an orbital shaker or a Biotek Synergy 2 plate reader at maximal shaking. Growth was recorded by optical density readings at 600 nm (OD_600_). For all growth experiments, overnight *B*. *subtilis* cultures in LB with antibiotics were diluted >1:50 in fresh prewarmed LB medium and grown for approximately 2.5 hours. This served as the pre-culture for all experiments with cells grown on LB medium. For experiments with cells grown on M9 medium, the LB pre-culture was subsequently diluted 1:20 in pre-warmed M9 medium and incubated for approximately 2.5 hours, which corresponds to mid- or early exponential growth. This culture then served as the pre-culture for experiments with cells grown on M9 medium. When required, media for *E*. *coli* were supplemented with ampicillin (100 μg ml^−1^) or chloramphenicol (10 μg ml^−1^); media for *B*. *subtilis* were supplemented with phleomycin (4 μg ml^−1^), kanamycin (20 μg ml^−1^), tetracyclin (5 μg ml^−1^), chloramphenicol (10 μg ml^−1^), erythromycin (2 μg ml^−1^), and spectinomycin (100 μg ml^−1^) or combinations thereof.

### Evolutionary conservation analysis of RnaC/S1022 targets

In order to find predicted targets co-conserved with RnaC/S1022, we used the 62 *Bacillus* genomes available in Genbank (as of January 31, 2013). On each of these genomes a BLAST search (Blastn v2.2.26 with default parameters) was conducted with the *B*. *subtilis* 168 RnaC/S1022 sequence as identified in Nicolas et al. [9]. Genomes where a homologue of RnaC/S1022 (E-value < 0.001) was found were then subjected to TargetRNA_v1 search with extended settings around the 5’UTR (−75 bp; +50 bp around the start codon and additional command line arguments “-z 250 -y 2 -l 6”) using as query the sequence of the first high-scoring-pair of the first BLAST hit in that particular genome. A bidirectional best hit criterion (based on Blastp v2.2.26 with default parameters and E-value cut-off 0.001) was used to compare the predicted targets in each genome with the predicted targets in the reference *B*. *subtilis* 168 genome (Genbank: AL009126-3). The data was tabulated and subsetted for *B*. *subtilis* 168 genes predicted for RnaC/S1022 in 8 or more genomes.

The *Bacillaceae* phylogenetic tree was computed based on an alignment of the *rpoB* gene BLAST result from the same set of genomes mentioned above. *RpoB* was reported to be a better determinant of evolutionary relatedness for *Bacillus* species than 16S rRNA [62].

### Western blot, RNA isolation and northern blot

Cultures grown on LB, M9G, or M9S were sampled in mid-exponential growth phase (OD_600_ 0.4–0.6) and were directly harvested in killing buffer and processed as previously described [9]. Northern blot analysis was carried out as described previously [63]. The digoxigenin-labeled RNA probe was synthesized by *in vitro* transcription with T7 RNA polymerase and an *abrB* specific PCR product as template. 5 μg of total RNA per lane was separated on 1.2% agarose gels. Chemiluminescence signals were detected using a ChemoCam Imager (Intas Science Image Instruments GmbH, Göttingen, Germany).

Western blot analysis was performed as described [64] using crude whole cell lysates. To prepare lysates, cell pellets were resuspended in LDS-sample buffer with reducing agent (Life technologies), and disrupted with glass beads in a bead beater (3 x 30 sec at 6500 rpm with 30 sec intermittences). Before loading on Novex nuPAGE 10% Bis-Tris gels (Life technologies), samples were boiled for 10 min and centrifuged to pellet the glass beads and cell debris. Equal OD units were loaded on gel and the intensity of the AbrB band was corrected with the intensity for the unrelated BdbD control.

Data from Northern blots and Western blots were quantified ImageJ software (available via http://rsbweb.nih.gov/ij/).

### Analysis of mRNA decay

Rifampicin (Sigma Aldrich) was added to 100 ml of exponentially growing M9G culture to a final concentration of 150 μg/ml from a 100x stock solution in methanol stored at −20°C. Just before the rifampicin addition and at 1, 2, 4, 6, 8 and 10 min after rifampicin addition, 10 ml of cells were harvested in killing buffer as described previously [9]. Cell pellets were washed once with 1 ml killing buffer and frozen in liquid nitrogen. RNA was extracted according to the hot phenol method as described previously [63]. Quantitative PCR was performed as described by Reilman et al. [51]. The Ct value corresponds to the PCR cycle at which the signal came above background.

We analyzed the four mRNA decay time-series (two strains and two replicates) with a non-linear model of mRNA concentration described in [65] that aims at capturing initial exponential decay followed by a plateau. The rate of the initial decay is supposed to correspond to the physiological degradation of the mRNA. In contrast, the final plateau can be contributed by several factors, such as background noise in measurement, a stable subpopulation of molecules, or a higher stability of the mRNAs at the end of the dynamic. In our context, we assumed that the mRNA concentration is proportional to 2^-Ct^ and thus we fitted (with the nls function of the R package stats) the model-C_t_(t_i_) = log_2_(A*(α_1_exp(-γ_1_t_i_)+α_2_)) + ε_i_, for i = 1…7 (t_i_ = 0, 1, 2, 4, 6, 8, 10 min) with A>0, α_1_>0, α_2_>0, γ_1_>0,α_1_+α_2_ = 0 and ε_i_ a Gaussian white noise. The estimates of the γ_1_ parameters of the first model were compared between the two genetic backgrounds (0 genomic copies vs. 2 copies of RnaC/S1022) with a student t-test after a log-transformation to stabilize the variance. For the 2-copy background, we also examined a second model that involves two exponential decay terms as would for instance arise when two sub-populations of mRNAs with distinct degradation rates coexist. It writes-C_t_(t_i_) = log_2_(A*(α_1_exp(-γ_1_t_i_)+α_2_exp(-γ_2_t_i_)+α_3_)) + ε_i_ with A>0, α_1_>0, α_2_>0, α_3_>0, γ_1_>γ_2_>0, and α_1_+α_2_+α_3_ = 0. For each pair of background and model, we plotted a “consensus” line whose parameters were obtained from the geometric mean between the two replicate experiments.

### Computation of promoter activity

Promoter activity was monitored every 10 min from cells grown in 96-well plates in a Biotek^®^ Synergy 2 plate reader. Promoter activity was computed by subtracting the fluorescence of the previous time-point from that of the measured time-point (as in Botella et al. [38]). Moving average filtering (*filter* function in R with filter = rep(1/5, 5) was applied for smoothing of the promoter activity plots.

### Flow cytometry and noise measurements

Cultures grown on LB, M9G, or M9S were sampled in mid-exponential growth phase OD_600_ 0.4–0.5 and were directly analyzed in an Accuri C6 flow cytometer. The number of recorded events within a gate set with growth medium was 15,000. The coefficient of variation (i.e. relative standard deviation) (CV%; standard deviation / mean * 100%) was used as a measure of the width of the distribution, or protein/promoter expression noise.

### Microscopy and live imaging

To inspect co-localization of AbrB-GFP with the nucleoid, cells were cultured until the exponential growth phase, pelleted by centrifugation, resuspended in 400μl phosphate-buffered saline (PBS) containing 1μl 500 ng/μl 4',6-diamidino-2-phenylindole (DAPI), and incubated for 10 min on ice. After this, the cells were washed once with PBS and slides were prepared for microscopy.

Live imaging analysis was conducted on aerated agarose cover slips as described previously [52]. Segmentation, calculation of Feret diameter, and auto-fluorescence correction for every microcolony were performed with ImageJ also as described by Piersma et al. [52]. Subsequent computations and plotting was done with R. The specific cell length (Feret diameter) increase per hour was computed as follows: ((cumulative Feret diameter at t_20 min_ / number of cells at t_0 min_) – (cumulative Feret diameter at t_0 min_ / number of cells at t_0 min_)) / ((t_20 min_—t_0 min_) / 60 min).

### Modeling noise

Noise promoting dynamics by sRNA regulation was modeled in a stochastic simulation model [45–47]. The considered reactions, employed parameters, and the master equation are listed in Fig. 6. The master equation was numerically integrated by employing an in-house developed implementation of the Gillespie algorithm [66] for each combination of model parameters. The stochastic simulations were started without any molecules and were run until a quasi-stationary state was reached. To capture the inherent stochasticity of the model we performed, for each set of model parameters, 50 x 10,000 simulation replicates (i.e. 500,000 in total). This can be interpreted as 50 experiments involving 10,000 cells each. Mean, standard deviation, and the median was computed for every molecular species in the population of 10,000 cells.

## Supporting Information

S1 Fig Sequence analysis of the RnaC/S1022 and *abrB* interaction. A) LocaRNA alignment of nine RnaC/S1022 sequences corresponding to the secondary structure predicted and shown in Fig. 1B. These sequences are derived from the *B*. *subtilis* genomes for which the interaction between RnaC/S1022 and *abrB* is predicted (genomes within the red box in Fig. 1A). Note that the diverging RnaC/S1022 sequence from the *B*. *atropheus* 1942 genome was excluded, because it would have added a large degree of uncertainty to the consensus structure presented in Fig. 1B, as is shown in panels E and F of this S1 Fig.. The mutated nucleotide that is essential for the interaction with *abrB* mRNA is indicated with an arrow. B) RNAfold [30] centroid structure based solely on the S1022 sequence from Nicolas et al. [28]. As indicated in the main text, this sequence is longer than that in Fig. 1B, but the predicted structure in the region that will interact with *abrB* is the same as the consensus sequence in Fig. 1B. The mutated nucleotide essential for the interaction with *abrB* mRNA is indicated with an arrow. C) T-COFFEE (http://www.tcoffee.org/) sequence alignment as visualized with Jalview (http://www.jalview.org/) of the *abrB* interaction region (-10 till +19 from the *B*. *subtilis* 168 *abrB* start codon) in all 19 species in which RnaC/S1022 is conserved (marked in the black box in Fig. 1A). The nucleotide essential for the interaction with RnaC/S1022 is indicated with an arrow and is conserved in all the genomes in which RnaC/S1022 is conserved. D) T-COFFEE alignment as visualized with Jalview of 19 conserved RnaC/S1022 sequences (genomes within the black box in Fig. 1A). The RnaC/S1022 hit from *B*. *atropheus* 1942 represents an in-between form of RnaC/S1022 since its sequence is most similar to that from the *B*. *amyloliquefaciens* sp. genomes while *abrB* is still predicted as a direct RnaC/S1022 target. The mutated nucleotide essential for the interaction with *abrB* mRNA is indicated with an arrow. E) LocaRNA sequence alignment of the same RnaC/S1022 sequences that were aligned in panel D. Due to the low level of similarity between these sequences no meaningful secondary structure can be predicted from this alignment as can be seen in panel E of this S1 Fig.. The mutated nucleotide essential for the interaction of *B*. *subtilis* 168 RnaC/S1022 with *abrB* mRNA is indicated with an arrow. F) LocaRNA structure prediction based on the RnaC/S1022 sequence alignment shown in panel E. This can be interpreted as a ‘nonsense structure’ due to the low level of similarity between the aligned sequences.
(TIF)

S2 Fig Growth phenotypes of cells lacking RnaC/S1022. The same growth curves as shown in Fig. 1C are presented as lin-log plots to distinguish between effects of the RnaC/S1022 mutation on growth rates and growth yields. When cells are grown on M9S, which results in the most drastic growth phenotype of RnaC/S1022 mutant cells, the growth rate is only slightly influenced by the RnaC/S1022 deletion while the growth yield is strongly increased.
(TIF)

S3 Fig Competence is decreased in an RnaC/S1022 mutant. Competence was assayed by transformation with plasmid pHB201. Error bars represent the standard deviation between three replicate experiments.
(TIF)

S4 Fig *abrB* mRNA is more stable in the absence of RnaC/S1022. Decreases in *abrB* mRNA levels after rifampicin addition were determined by qPCR using equal amounts of RNA per strain and time-point. The RNA from the RnaC/S1022 deletion strain (blue symbols) was compared with RNA from the strain with two chromosomal RnaC/S1022 copies (green symbols). Two non-linear models were fitted to these data: a first model with a single decay rate followed by a plateau (fit illustrated with plain line); a second model with two decay rates (fit illustrated with interrupted line). The initial decay rate (as estimated by the γ_1_ parameter of the first model) was significantly higher in the strain with two copies of RnaC/S1022 (Students t-test, p-value <0.05). The second model provided a better fit to the data of the strain with two genomic copies of RnaC/S1022, and leads to even higher estimates of the initial decay rate (theγ_1_ parameter of the second model).
(TIF)

S5 Fig AbrB-GFP localizes to the nucleoid. Fluorescence microscopy images of the Δ*spo0A* AbrB-GFP strain (left panels) and the *amyE*::P_spac_ GFP strain (right panels). DAPI was used to stain the DNA. As shown in the image overlay, AbrB-GFP fluorescence colocalizes with the DAPI-stained nucleoid as expected from the fact that AbrB is a DNA-binding protein.
(TIF)

S6 Fig *B*. *subtilis* Hfq has no effect on expression of the AbrB-GFP reporter. Representative FC histograms of AbrB-GFP expression by cells of the parental *B*. *subtilis* strain 168, a Δ*hfq* mutant, and a Δ*hfq* ΔRnaC/S1022 double mutant grown on M9G. The profile of AbrB-GFP expression in the *hfq* mutant strain is identical to that in the parental strain, indicating that Hfq has no role in mediating the direct interaction between RnaC/S1022 and *abrB*.
(TIF)

S7 Fig AbrB does not regulate the RnaC/S1022 promoter. Representative FC histograms of the parental strain *B*. *subtilis* 168 and the Δ*abrB* strain carrying the P_RnaC/S1022_-*gfp* construct. These experiments were performed with cells grown on LB, because of the M9G/M9S growth phenotypes of *abrB* mutant strains.
(TIF)

S8 Fig Modeling outcomes for both sets of parameters at variable extrinsic noise levels. Modeling outcomes as described in Fig. 6 for all five considered intrinsic noise levels obtained with parameter Sets I and II. Different intrinsic noise levels are marked with differently colored symbols.
(TIF)

S9 Fig Growth and AbrB-GFP levels of colonies from Fig. 8C and the S1 Movie. Cell growth is expressed as the cumulative cell length (Feret’s diameter). Cell #1 with a higher initial AbrB-GFP level grows faster than cell #2 with a lower initial AbrB-GFP level.
(TIF)

S1 Table Conserved predicted targets of RnaC/S1022. (XLSX)

S2 Table Strains used in this study. (XLSX)

S3 Table Primers used in this study. (XLSX)

S1 Movie Original movie of the two adjacent cells shown in Fig. 8C and S8 Fig.
The white outlines mark the contours of the cell.
(AVI)

## References

[1] Buescher JM, Liebermeister W, Jules M, Uhr M, Muntel J, et al (2012) Global network reorganization during dynamic adaptations of *Bacillus subtilis* metabolism. Science 335(6072): 1099–1103. 10.1126/science.1206871 22383848

[2] Beisel CL, Storz G. (2010) Base pairing small RNAs and their roles in global regulatory networks. FEMS Microbiol Rev 34(5): 866–882. 10.1111/j.1574-6976.2010.00241.x 20662934 PMC2920360

[3] Storz G, Vogel J, Wassarman KM. (2011) Regulation by small RNAs in bacteria: Expanding frontiers. Mol Cell 43(6): 880–891. 10.1016/j.molcel.2011.08.022 21925377 PMC3176440

[4] Aiba H. (2007) Mechanism of RNA silencing by Hfq-binding small RNAs. Curr Opin Microbiol 10(2): 134–139. 17383928 10.1016/j.mib.2007.03.010

[5] Gaballa A, Antelmann H, Aguilar C, Khakh SK, Song KB, et al (2008) The *Bacillus subtilis* iron-sparing response is mediated by a Fur-regulated small RNA and three small, basic proteins. Proc Natl Acad Sci U S A 105(33): 11927–11932. 10.1073/pnas.0711752105 18697947 PMC2575260

[6] Heidrich N, Chinali A, Gerth U, Brantl S. (2006) The small untranslated RNA SR1 from the *Bacillus subtilis* genome is involved in the regulation of arginine catabolism. Mol Microbiol 62(2): 520–536. 17020585 10.1111/j.1365-2958.2006.05384.x

[7] Smaldone GT, Revelles O, Gaballa A, Sauer U, Antelmann H, et al (2012) A global investigation of the *Bacillus subtilis* iron-sparing response identifies major changes in metabolism. J Bacteriol 194(10): 2594–2605. 10.1128/JB.05990-11 22389480 PMC3347201

[8] Irnov I, Sharma CM, Vogel J, Winkler WC. (2010) Identification of regulatory RNAs in *Bacillus subtilis* . Nucleic Acids Res 38(19): 6637–6651. 10.1093/nar/gkq454 20525796 PMC2965217

[9] Nicolas P, Mader U, Dervyn E, Rochat T, Leduc A, et al (2012) Condition-dependent transcriptome reveals high-level regulatory architecture in *Bacillus subtilis* . Science 335(6072): 1103–1106. 10.1126/science.1206848 22383849

[10] Raj A, van Oudenaarden A. (2008) Nature, nurture, or chance: Stochastic gene expression and its consequences. Cell 135(2): 216–226. 10.1016/j.cell.2008.09.050 18957198 PMC3118044

[11] Losick R, Desplan C. (2008) Stochasticity and cell fate. Science 320(5872): 65–68. 10.1126/science.1147888 18388284 PMC2605794

[12] Maamar H, Raj A, Dubnau D. (2007) Noise in gene expression determines cell fate in *Bacillus subtilis* . Science 317(5837): 526–529. 17569828 10.1126/science.1140818 PMC3828679

[13] Chastanet A, Vitkup D, Yuan GC, Norman TM, Liu JS, et al (2010) Broadly heterogeneous activation of the master regulator for sporulation in *Bacillus subtilis* . Proc Natl Acad Sci U S A 107(18): 8486–8491. 10.1073/pnas.1002499107 20404177 PMC2889527

[14] Veening JW, Smits WK, Kuipers OP. (2008) Bistability, epigenetics, and bet-hedging in bacteria. Annu Rev Microbiol 62: 193–210. 10.1146/annurev.micro.62.081307.163002 18537474

[15] Elowitz MB, Levine AJ, Siggia ED, Swain PS. (2002) Stochastic gene expression in a single cell. Science 297(5584): 1183–1186. 12183631 10.1126/science.1070919

[16] Paulsson J. (2004) Summing up the noise in gene networks. Nature 427(6973): 415–418. 14749823 10.1038/nature02257

[17] Balaban NQ, Merrin J, Chait R, Kowalik L, Leibler S. (2004) Bacterial persistence as a phenotypic switch. Science 305(5690): 1622–1625. 15308767 10.1126/science.1099390

[18] Eldar A, Elowitz MB. (2010) Functional roles for noise in genetic circuits. Nature 467(7312): 167–173. 10.1038/nature09326 20829787 PMC4100692

[19] Madar D, Dekel E, Bren A, Alon U. (2011) Negative auto-regulation increases the input dynamic-range of the arabinose system of *Escherichia coli* . BMC Syst Biol 5: 111–0509–5–111. 10.1186/1752-0509-5-111.Negative 21749723 PMC3163201

[20] Saile E, Koehler TM. (2002) Control of anthrax toxin gene expression by the transition state regulator *abrB* . J Bacteriol 184(2): 370–380. 11751813 10.1128/JB.184.2.370-380.2002 PMC139583

[21] Glaser P, Frangeul L, Buchrieser C, Rusniok C, Amend A, et al (2001) Comparative genomics of *Listeria* species. Science 294(5543): 849–852. 11679669 10.1126/science.1063447

[22] Fisher SH, Strauch MA, Atkinson MR, Wray LV Jr. (1994) Modulation of *Bacillus subtilis* catabolite repression by transition state regulatory protein AbrB. J Bacteriol 176(7): 1903–1912. 8144456 10.1128/jb.176.7.1903-1912.1994 PMC205293

[23] Mader U, Schmeisky AG, Florez LA, Stulke J. (2012) SubtiWiki—a comprehensive community resource for the model organism *Bacillus subtilis* . Nucleic Acids Res 40(Database issue): D1278–87. 10.1093/nar/gkr923 22096228 PMC3245094

[24] Chumsakul O, Takahashi H, Oshima T, Hishimoto T, Kanaya S, et al (2011) Genome-wide binding profiles of the *Bacillus subtilis* transition state regulator AbrB and its homolog Abh reveals their interactive role in transcriptional regulation. Nucleic Acids Res 39(2): 414–428. 10.1093/nar/gkq780 20817675 PMC3025583

[25] Bobay BG, Benson L, Naylor S, Feeney B, Clark AC, et al (2004) Evaluation of the DNA binding tendencies of the transition state regulator AbrB. Biochemistry 43(51): 16106–16118. 15610005 10.1021/bi048399h

[26] Banse AV, Chastanet A, Rahn-Lee L, Hobbs EC, Losick R. (2008) Parallel pathways of repression and antirepression governing the transition to stationary phase in *Bacillus subtilis* . Proc Natl Acad Sci U S A 105(40): 15547–15552. 10.1073/pnas.0805203105 18840696 PMC2563134

[27] Schultz D, Wolynes PG, Ben Jacob E, Onuchic JN. (2009) Deciding fate in adverse times: Sporulation and competence in *Bacillus subtilis* . Proc Natl Acad Sci U S A 106(50): 21027–21034. 10.1073/pnas.0912185106 19995980 PMC2795487

[28] Schmalisch M, Maiques E, Nikolov L, Camp AH, Chevreux B, et al (2010) Small genes under sporulation control in the *Bacillus subtilis* genome. J Bacteriol 192(20): 5402–5412. 10.1128/JB.00534-10 20709900 PMC2950494

[29] Will S, Joshi T, Hofacker IL, Stadler PF, Backofen R. (2012) LocARNA-P: Accurate boundary prediction and improved detection of structural RNAs. RNA 18(5): 900–914. 10.1261/rna.029041.111 22450757 PMC3334699

[30] Hofacker IL, Stadler PF. (2006) Memory efficient folding algorithms for circular RNA secondary structures. Bioinformatics 22(10): 1172–1176. 16452114 10.1093/bioinformatics/btl023

[31] Nicholson WL. (2012) Increased competitive fitness of *Bacillus subtilis* under nonsporulating conditions via inactivation of pleiotropic regulators AlsR, SigD, and SigW. Appl Environ Microbiol 78(9): 3500–3503. 10.1128/AEM.07742-11 22344650 PMC3346490

[32] Tjaden B. (2008) TargetRNA: A tool for predicting targets of small RNA action in bacteria. Nucleic Acids Res 36(Web Server issue): W109–13. 10.1093/nar/gkn264 18477632 PMC2447797

[33] Horsburgh MJ, Moir A. (1999) Sigma M, an ECF RNA polymerase sigma factor of *Bacillus subtilis* 168, is essential for growth and survival in high concentrations of salt. Mol Microbiol 32(1): 41–50. 10216858 10.1046/j.1365-2958.1999.01323.x

[34] Xu K, Clark D, Strauch MA. (1996) Analysis of *abrB* mutations, mutant proteins, and why *abrB* does not utilize a perfect consensus in the-35 region of its sigma A promoter. J Biol Chem 271(5): 2621–2626. 8576231 10.1074/jbc.271.5.2621

[35] Hahn J, Roggiani M, Dubnau D. (1995) The major role of Spo0A in genetic competence is to downregulate *abrB*, an essential competence gene. J Bacteriol 177(12): 3601–3605. 7768874 10.1128/jb.177.12.3601-3605.1995 PMC177070

[36] Deana A, Belasco JG. (2005) Lost in translation: The influence of ribosomes on bacterial mRNA decay. Genes Dev 19(21): 2526–2533. 16264189 10.1101/gad.1348805

[37] Peer A, Margalit H. (2011) Accessibility and evolutionary conservation mark bacterial small-RNA target-binding regions. J Bacteriol 193(7): 1690–1701. 10.1128/JB.01419-10 21278294 PMC3067639

[38] Botella E, Fogg M, Jules M, Piersma S, Doherty G, et al (2010) pBaSysBioII: An integrative plasmid generating *gfp* transcriptional fusions for high-throughput analysis of gene expression in *Bacillus subtilis* . Microbiology 156(Pt 6): 1600–1608. 10.1099/mic.0.035758-0 20150235

[39] Doherty GP, Fogg MJ, Wilkinson AJ, Lewis PJ. (2010) Small subunits of RNA polymerase: Localization, levels and implications for core enzyme composition. Microbiology 156(Pt 12): 3532–3543. 10.1099/mic.0.041566-0 20724389

[40] Smits WK, Kuipers OP, Veening JW. (2006) Phenotypic variation in bacteria: The role of feedback regulation. Nat Rev Microbiol 4(4): 259–271. 16541134 10.1038/nrmicro1381

[41] Greene EA, Spiegelman GB. (1996) The Spo0A protein of *Bacillus subtilis* inhibits transcription of the *abrB* gene without preventing binding of the polymerase to the promoter. J Biol Chem 271(19): 11455–11461. 8626703 10.1074/jbc.271.19.11455

[42] Becskei A, Serrano L. (2000) Engineering stability in gene networks by autoregulation. Nature 405(6786): 590–593. 10850721 10.1038/35014651

[43] Dublanche Y, Michalodimitrakis K, Kummerer N, Foglierini M, Serrano L. (2006) Noise in transcription negative feedback loops: Simulation and experimental analysis. Mol Syst Biol 2: 41 16883354 10.1038/msb4100081 PMC1681513

[44] Kearns DB, Losick R. (2005) Cell population heterogeneity during growth of *Bacillus subtilis* . Genes Dev 19(24): 3083–3094. 16357223 10.1101/gad.1373905 PMC1315410

[45] Levine E, Zhang Z, Kuhlman T, Hwa T. (2007) Quantitative characteristics of gene regulation by small RNA. PLoS Biol 5(9): e229 17713988 10.1371/journal.pbio.0050229 PMC1994261

[46] Arbel-Goren R, Tal A, Friedlander T, Meshner S, Costantino N, et al (2013) Effects of post-transcriptional regulation on phenotypic noise in *Escherichia coli* . Nucleic Acids Res 41(9): 4825–4834. 10.1093/nar/gkt184 23519613 PMC3643596

[47] Jia Y, Liu W, Li A, Yang L, Zhan X. (2009) Intrinsic noise in post-transcriptional gene regulation by small non-coding RNA. Biophys Chem 143(1–2): 60–69. 10.1016/j.bpc.2009.05.003 19403234

[48] Hambraeus G, von Wachenfeldt C, Hederstedt L. (2003) Genome-wide survey of mRNA half-lives in *Bacillus subtilis* identifies extremely stable mRNAs. Mol Genet Genomics 269(5): 706–714. 12884008 10.1007/s00438-003-0883-6

[49] Jost D, Nowojewski A, Levine E. (2013) Regulating the many to benefit the few: Role of weak small RNA targets. Biophys J 104(8): 1773–1782. 10.1016/j.bpj.2013.02.020 23601324 PMC3627871

[50] Britton RA, Eichenberger P, Gonzalez-Pastor JE, Fawcett P, Monson R, et al (2002) Genome-wide analysis of the stationary-phase sigma factor (sigma-H) regulon of *Bacillus subtilis* . J Bacteriol 184(17): 4881–4890. 12169614 10.1128/JB.184.17.4881-4890.2002 PMC135291

[51] Reilman E, Mars RA, van Dijl JM, Denham EL. (2015) The multidrug ABC transporter BmrC/BmrD of *Bacillus subtilis* is regulated via a ribosome-mediated transcriptional attenuation mechanism. Nucleic Acids Res 42(18): 11393–11407. 10.1093/nar/gku832 PMC4191407 25217586

[52] Piersma S, Denham EL, Drulhe S, Tonk RH, Schwikowski B, et al (2013) TLM-quant: An open-source pipeline for visualization and quantification of gene expression heterogeneity in growing microbial cells. PLoS One 8(7): e68696 10.1371/journal.pone.0068696 23874729 PMC3714294

[53] Gefen O, Balaban NQ. (2009) The importance of being persistent: Heterogeneity of bacterial populations under antibiotic stress. FEMS Microbiol Rev 33(4): 704–717. 10.1111/j.1574-6976.2008.00156.x 19207742

[54] Tuomanen E, Cozens R, Tosch W, Zak O, Tomasz A. (1986) The rate of killing of *Escherichia coli* by ß-lactam antibiotics is strictly proportional to the rate of bacterial growth. Journal of General Microbiology 132(5): 1297–1304. 3534137 10.1099/00221287-132-5-1297

[55] Hecker M, Pane-Farre J, Volker U. (2007) SigB-dependent general stress response in *Bacillus subtilis* and related Gram-positive bacteria. Annu Rev Microbiol 61: 215–236. 18035607 10.1146/annurev.micro.61.080706.093445

[56] Rotem E, Loinger A, Ronin I, Levin-Reisman I, Gabay C, et al (2010) Regulation of phenotypic variability by a threshold-based mechanism underlies bacterial persistence. Proc Natl Acad Sci U S A 107(28): 12541–12546. 10.1073/pnas.1004333107 20616060 PMC2906590

[57] Kunst F, Rapoport G. (1995) Salt stress is an environmental signal affecting degradative enzyme synthesis in *Bacillus subtilis* . J Bacteriol 177(9): 2403–2407. 7730271 10.1128/jb.177.9.2403-2407.1995 PMC176898

[58] Tanaka K, Henry CS, Zinner JF, Jolivet E, Cohoon MP, et al (2013) Building the repertoire of dispensable chromosome regions in *Bacillus subtilis* entails major refinement of cognate large-scale metabolic model. Nucleic Acids Res 41(1): 687–699. 10.1093/nar/gks963 23109554 PMC3592452

[59] Darmon E, Dorenbos R, Meens J, Freudl R, Antelmann H, et al (2006) A disulfide bond-containing alkaline phosphatase triggers a BdbC-dependent secretion stress response in *Bacillus subtilis* . Appl Environ Microbiol 72(11): 6876–6885. 17088376 10.1128/AEM.01176-06 PMC1636209

[60] Quan J, Tian J. (2009) Circular polymerase extension cloning of complex gene libraries and pathways. PLoS One 4(7): e6441 10.1371/journal.pone.0006441 19649325 PMC2713398

[61] Haun RS, Serventi IM, Moss J. (1992) Rapid, reliable ligation-independent cloning of PCR products using modified plasmid vectors. BioTechniques 13(4): 515–518. 1362067

[62] Ki JS, Zhang W, Qian PY. (2009) Discovery of marine bacillus species by 16S rRNA and *rpoB* comparisons and their usefulness for species identification. J Microbiol Methods 77(1): 48–57. 10.1016/j.mimet.2009.01.003 19166882

[63] Homuth G, Masuda S, Mogk A, Kobayashi Y, Schumann W. (1997) The *dnaK* operon of *Bacillus subtilis* is heptacistronic. J Bacteriol 179(4): 1153–1164. 9023197 10.1128/jb.179.4.1153-1164.1997 PMC178811

[64] Zweers JC, Wiegert T, van Dijl JM. (2009) Stress-responsive systems set specific limits to the overproduction of membrane proteins in *Bacillus subtilis* . Appl Environ Microbiol 75(23): 7356–7364. 10.1128/AEM.01560-09 19820159 PMC2786430

[65] Shock JL, Fischer KF, DeRisi JL. (2007) Whole-genome analysis of mRNA decay in *Plasmodium falciparum* reveals a global lengthening of mRNA half-life during the intra-erythrocytic development cycle. Genome Biol 8(7): R134 17612404 10.1186/gb-2007-8-7-r134 PMC2323219

[66] Gillespie DT. (1977) Exact stochastic simulation of coupled chemical reactions. J Phys Chem 81(25): 2340–2361.

