# Supplementary figures and images for: Small Regulatory RNA-Induced Growth Rate Heterogeneity of Bacillus subtilis
Source: PLoS Genet. 2015 Mar 19;11(3):e1005046. doi: 10.1371/journal.pgen.1005046 (PMC4366234; doi:10.1371/journal.pgen.1005046)

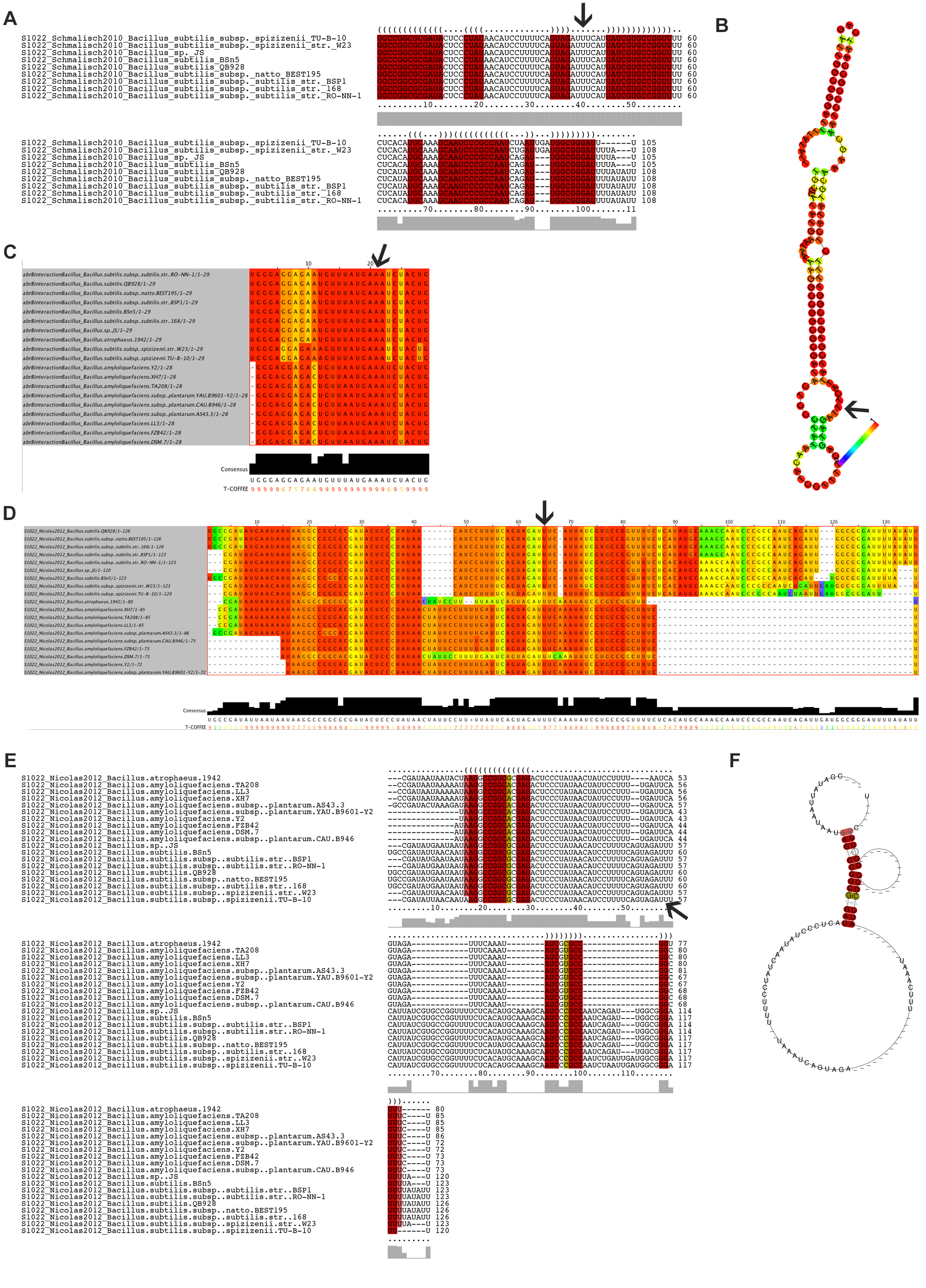

Supplement: S1 Fig — A) LocaRNA alignment of nine RnaC/S1022 sequences corresponding to the secondary structure predicted and shown in Fig. 1B. These sequences are derived from the B. subtilis genomes for which the interaction between RnaC/S1022 and abrB is predicted (genomes within the red box in Fig. 1A). Note that the diverging RnaC/S1022 sequence from the B. atropheus 1942 genome was excluded, because it would have added a large degree of uncertainty to the consensus structure presented in Fig. 1B, as is shown in panels E and F of this S1 Fig.. The mutated nucleotide that is essential for the interaction with abrB mRNA is indicated with an arrow. B) RNAfold [30] centroid structure based solely on the S1022 sequence from Nicolas et al. [28]. As indicated in the main text, this sequence is longer than that in Fig. 1B, but the predicted structure in the region that will interact with abrB is the same as the consensus sequence in Fig. 1B. The mutated nucleotide essential for the interaction with abrB mRNA is indicated with an arrow. C) T-COFFEE (http://www.tcoffee.org/) sequence alignment as visualized with Jalview (http://www.jalview.org/) of the abrB interaction region (-10 till +19 from the B. subtilis 168 abrB start codon) in all 19 species in which RnaC/S1022 is conserved (marked in the black box in Fig. 1A). The nucleotide essential for the interaction with RnaC/S1022 is indicated with an arrow and is conserved in all the genomes in which RnaC/S1022 is conserved. D) T-COFFEE alignment as visualized with Jalview of 19 conserved RnaC/S1022 sequences (genomes within the black box in Fig. 1A). The RnaC/S1022 hit from B. atropheus 1942 represents an in-between form of RnaC/S1022 since its sequence is most similar to that from the B. amyloliquefaciens sp. genomes while abrB is still predicted as a direct RnaC/S1022 target. The mutated nucleotide essential for the interaction with abrB mRNA is indicated with an arrow. E) LocaRNA sequence alignment of the same RnaC/S1022 sequences that we [file pgen.1005046.s001.tif]

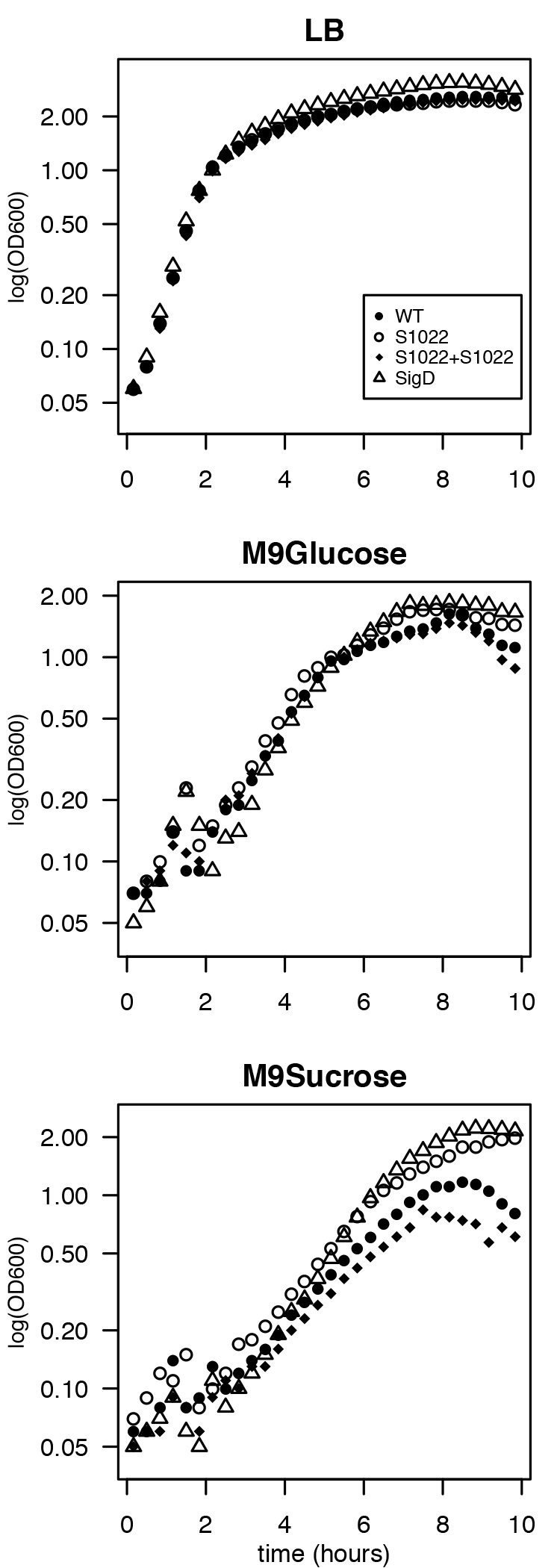

Supplement: S2 Fig — The same growth curves as shown in Fig. 1C are presented as lin-log plots to distinguish between effects of the RnaC/S1022 mutation on growth rates and growth yields. When cells are grown on M9S, which results in the most drastic growth phenotype of RnaC/S1022 mutant cells, the growth rate is only slightly influenced by the RnaC/S1022 deletion while the growth yield is strongly increased. (TIF) [file pgen.1005046.s002.tif]

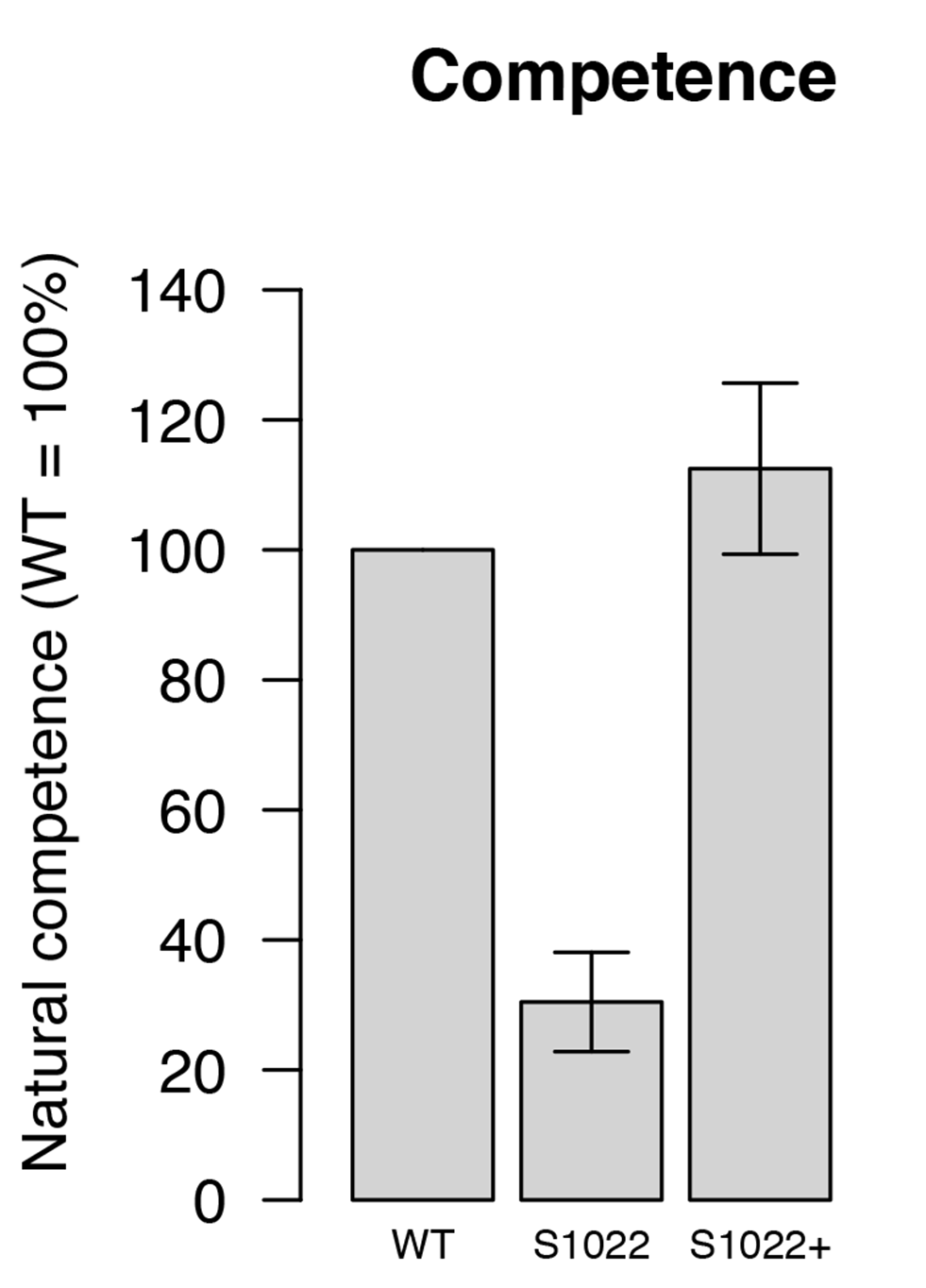

Supplement: S3 Fig — Competence was assayed by transformation with plasmid pHB201. Error bars represent the standard deviation between three replicate experiments. (TIF) [file pgen.1005046.s003.tif]

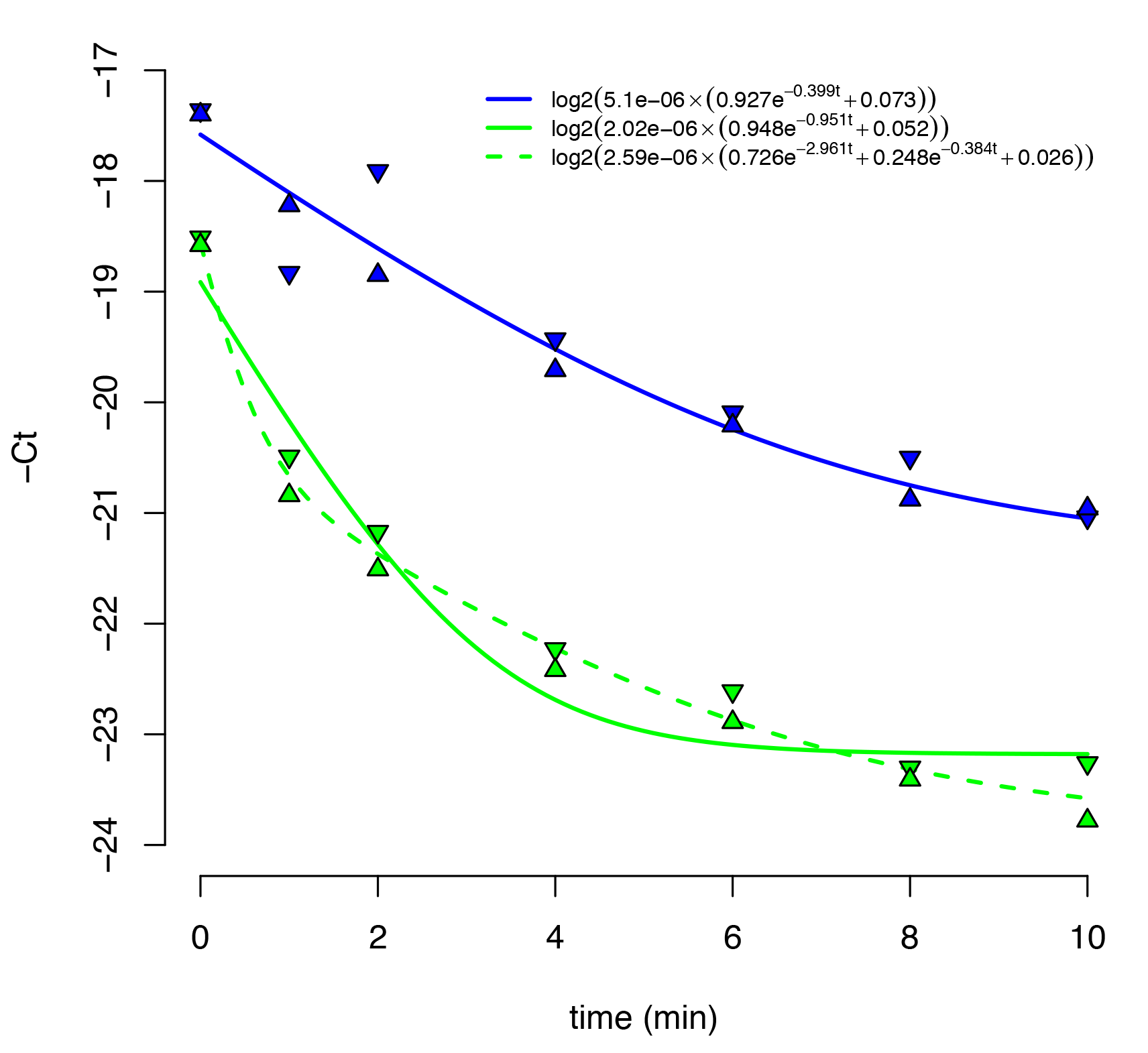

Supplement: S4 Fig — Decreases in abrB mRNA levels after rifampicin addition were determined by qPCR using equal amounts of RNA per strain and time-point. The RNA from the RnaC/S1022 deletion strain (blue symbols) was compared with RNA from the strain with two chromosomal RnaC/S1022 copies (green symbols). Two non-linear models were fitted to these data: a first model with a single decay rate followed by a plateau (fit illustrated with plain line); a second model with two decay rates (fit illustrated with interrupted line). The initial decay rate (as estimated by the γ1 parameter of the first model) was significantly higher in the strain with two copies of RnaC/S1022 (Students t-test, p-value <0.05). The second model provided a better fit to the data of the strain with two genomic copies of RnaC/S1022, and leads to even higher estimates of the initial decay rate (theγ1 parameter of the second model). (TIF) [file pgen.1005046.s004.tif]

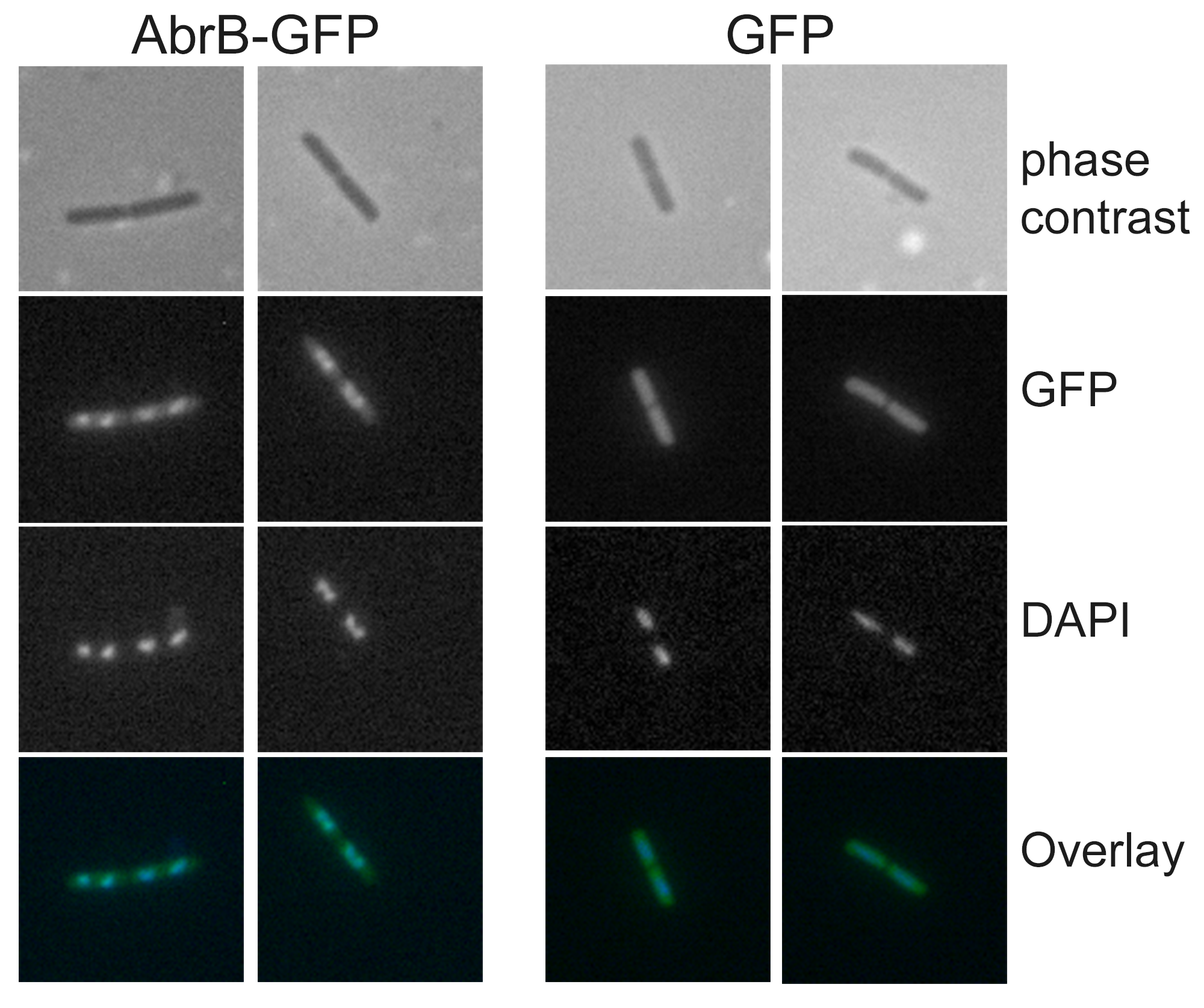

Supplement: S5 Fig — Fluorescence microscopy images of the Δspo0A AbrB-GFP strain (left panels) and the amyE::Pspac GFP strain (right panels). DAPI was used to stain the DNA. As shown in the image overlay, AbrB-GFP fluorescence colocalizes with the DAPI-stained nucleoid as expected from the fact that AbrB is a DNA-binding protein. (TIF) [file pgen.1005046.s005.tif]

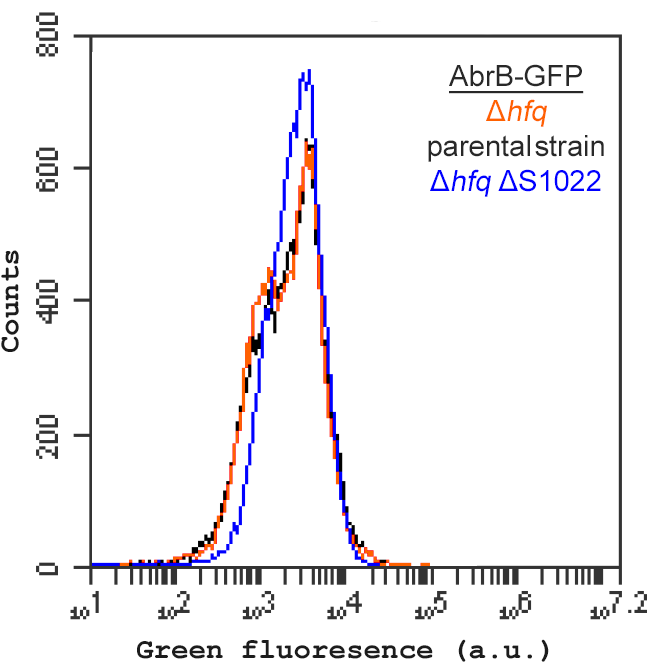

Supplement: S6 Fig — Representative FC histograms of AbrB-GFP expression by cells of the parental B. subtilis strain 168, a Δhfq mutant, and a Δhfq ΔRnaC/S1022 double mutant grown on M9G. The profile of AbrB-GFP expression in the hfq mutant strain is identical to that in the parental strain, indicating that Hfq has no role in mediating the direct interaction between RnaC/S1022 and abrB. (TIF) [file pgen.1005046.s006.tif]

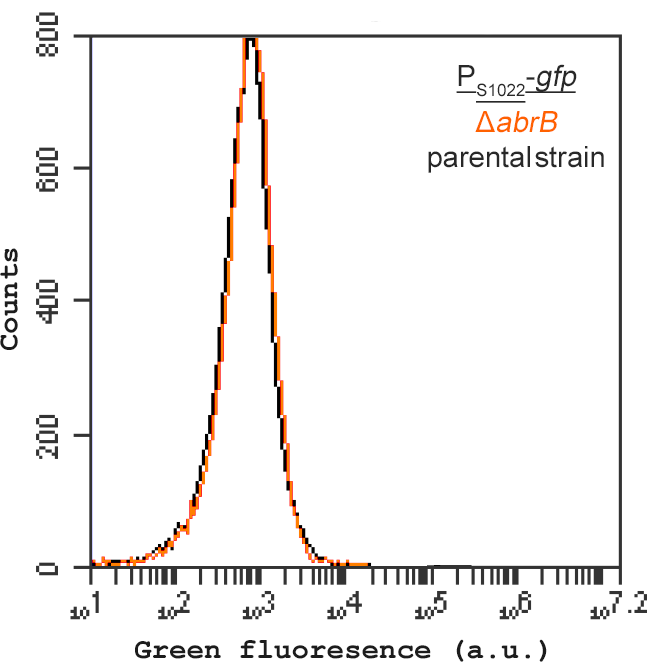

Supplement: S7 Fig — Representative FC histograms of the parental strain B. subtilis 168 and the ΔabrB strain carrying the PRnaC/S1022-gfp construct. These experiments were performed with cells grown on LB, because of the M9G/M9S growth phenotypes of abrB mutant strains. (TIF) [file pgen.1005046.s007.tif]

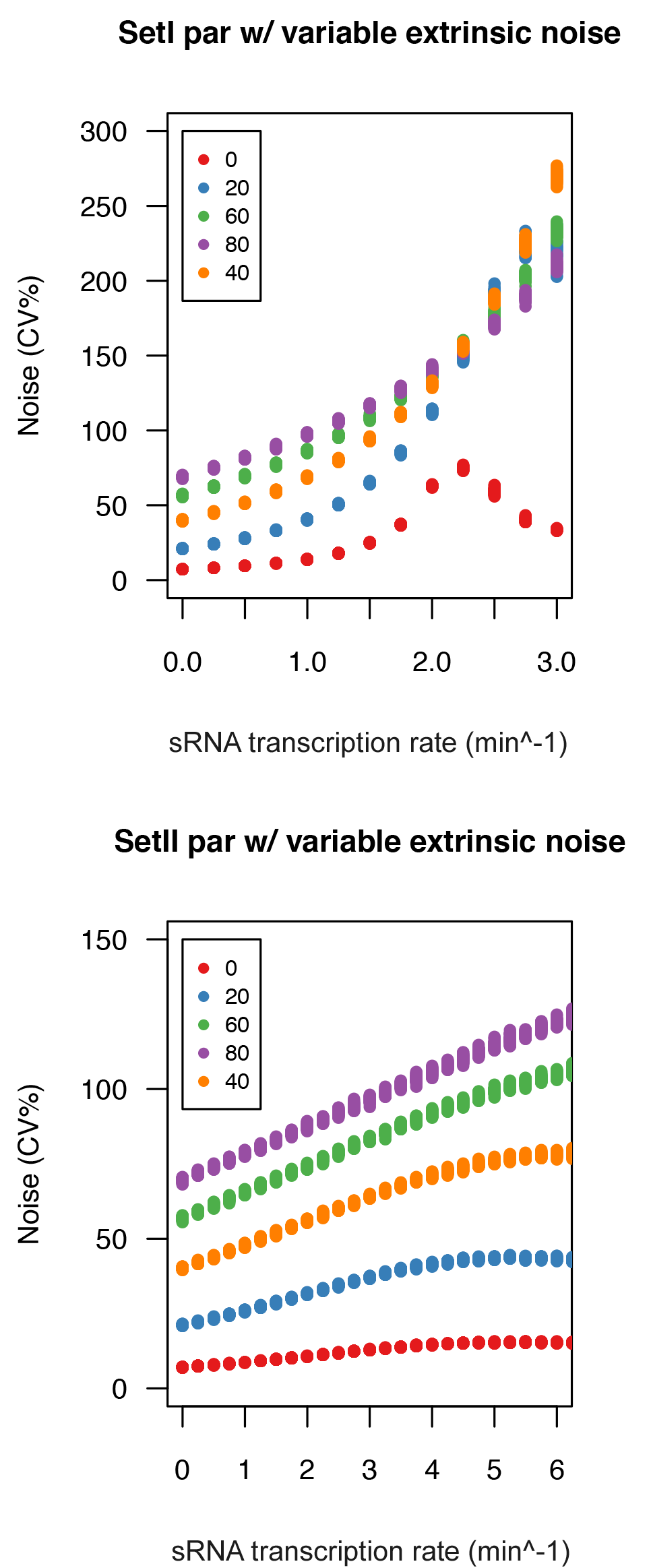

Supplement: S8 Fig — Modeling outcomes as described in Fig. 6 for all five considered intrinsic noise levels obtained with parameter Sets I and II. Different intrinsic noise levels are marked with differently colored symbols. (TIF) [file pgen.1005046.s008.tif]

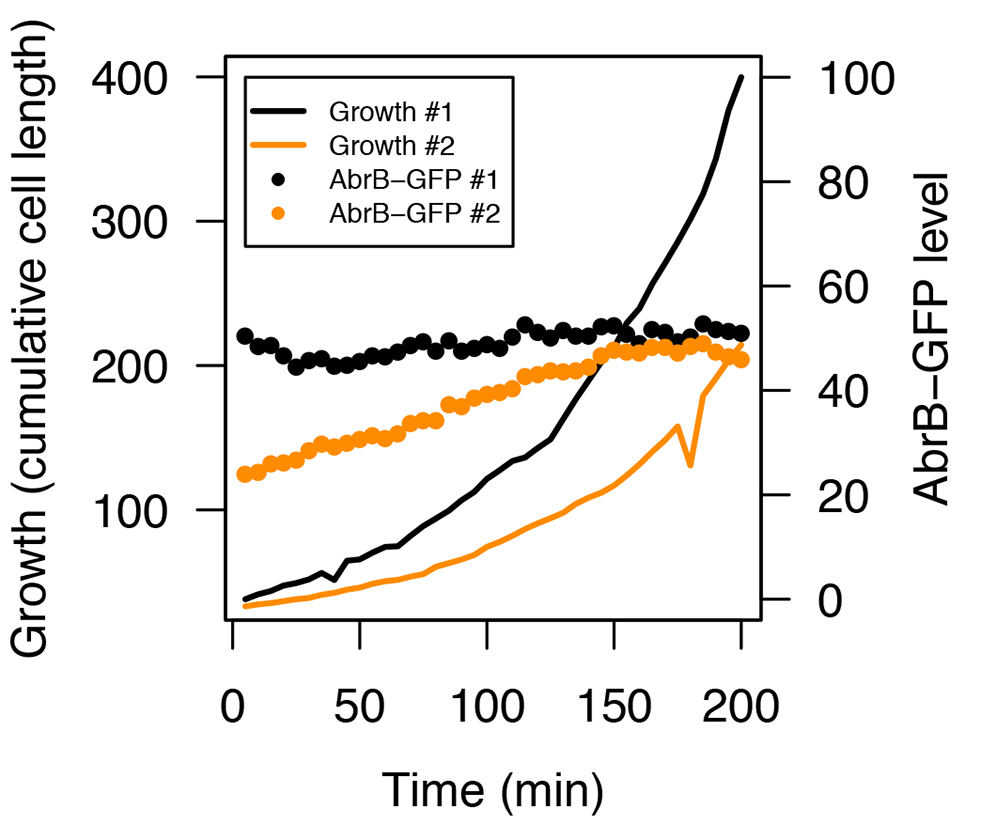

Supplement: S9 Fig — Cell growth is expressed as the cumulative cell length (Feret’s diameter). Cell #1 with a higher initial AbrB-GFP level grows faster than cell #2 with a lower initial AbrB-GFP level. (TIF) [file pgen.1005046.s009.tif]
